# Molecular evolutionary analysis of the *SHI/STY* gene family in land plants: A focus on the *Brassica* species

**DOI:** 10.3389/fpls.2022.958964

**Published:** 2022-08-04

**Authors:** Da Fang, Weimeng Zhang, Xiuzhu Cheng, Fei Hu, Ziyi Ye, Jun Cao

**Affiliations:** School of Life Sciences, Jiangsu University, Zhenjiang, Jiangsu, China

**Keywords:** *SHI/STY* gene family, molecular evolution, gene duplication, *Cis*-acting elements, land plants

## Abstract

The plant-specific SHORT INTERNODES/STYLISH (SHI/STY) proteins belong to a family of transcription factors that are involved in the formation and development of early lateral roots. However, the molecular evolution of this family is rarely reported. Here, a total of 195 *SHI/STY* genes were identified in 21 terrestrial plants, and the *Brassica* species is the focus of our research. Their physicochemical properties, chromosome location and duplication, motif distribution, exon-intron structures, genetic evolution, and expression patterns were systematically analyzed. These genes are divided into four clades (Clade 1/2/3/4) based on phylogenetic analysis. Motif distribution and gene structure are similar in each clade. SHI/STY proteins are localized in the nucleus by the prediction of subcellular localization. Collinearity analysis indicates that the *SHI/STYs* are relatively conserved in evolution. Whole-genome duplication is the main factor for their expansion. *SHI/STYs* have undergone intense purifying selection, but several positive selection sites are also identified. Most promoters of *SHI/STY* genes contain different types of *cis*-elements, such as light, stress, and hormone-responsive elements, suggesting that they may be involved in many biological processes. Protein–protein interaction predicted some important SHI/STY interacting proteins, such as LPAT4, MBOATs, PPR, and UBQ3. In addition, the RNA-seq and qRT-PCR analysis were studied in detail in rape. As a result, *SHI/STYs* are highly expressed in root and bud, and can be affected by *Sclerotinia sclerotiorum*, drought, cold, and heat stresses. Moreover, quantitative real-time PCR (qRT-PCR) analyses indicates that expression levels of *BnSHI/STYs* are significantly altered in different treatments (cold, salt, drought, IAA, auxin; ABA, abscisic acid; 6-BA, cytokinin). It provides a new understanding of the evolution and expansion of the *SHI/STY* family in land plants and lays a foundation for further research on their functions.

## Introduction

Gene families play important roles in the evolution of plants, but their functions and evolutionary history are not well understood. Ancient SHORT INTERNODES/STYLISH (SHI/STY) protein family of transcription factors have been found in all sequenced land plant species, from *Physcomitrella patens* to flowering plants, like *Arabidopsis thaliana*, *Brassica napus*, *Zea mays,* and *Glycine max*. The extensive presence of these proteins in terrestrial plants suggests that they may participate in important functions for plant growth and development (Kuusk et al., 2006). In *Arabidopsis*, ten *SHI/STY* members were reported, containing *SHI*, *STY1*, *STY2*, *LATERAL ROOT PRIMORDIUM1* (*LRP1*), *SHI RELATED SEQUENCE3* (*SRS3*), *SRS4*, *SRS5*, *SRS6*, *SRS7*, *SRS8* (Singh et al., 2020). They have a highly conserved IGGH domain that covers a RING-like zinc finger domain (CX2CX7CX4CX2C2X6C) for binding to RNA, protein, and lipid substrates (Fridborg et al., 2001; Kuusk et al., 2006). The zinc finger domain is composed of one or several small protein motifs, which can contact the target molecules by forming stable finger-like projections (Klug, 1999; Laity et al., 2001).

The cysteine arrangement in SHI/STYs (about 111–139 amino acids), H-XI2-C-XX2C-Xlo-14-C-X2-C-X4-H-X2-C-X ~ -C or C-X2-CXI2-C-X2-C-X7-C (the X could be any amino acid), is conserved in the Histidine and Cysteine domain of the Zinc-binding site of protein kinase C in their activation domain (Hubbard et al., 1991). Furthermore, the IGGH domain of the SHI/STY proteins contains acidic amino acids and can act as a transcriptional activator (Fridborg et al., 2001; Singh et al., 2020).

*SHI/STY* genes not only regulate the growth and development of plants, but also respond to hormones and some abiotic stresses (Zhao et al., 2020). Several studies have shown that *SHI/STY* genes are associated with auxin signaling pathways in terrestrial plants and play important functions in the regulation of plants tissues and other hormonal pathways (Eklund et al., 2010; Zawaski et al., 2011; Islam et al., 2013; Youssef et al., 2017). In addition, some members are involved in auxin biosynthesis by regulating genes related to photomorphogenesis (Eklund et al., 2010; Staldal et al., 2012; Baylis et al., 2013; Yuan et al., 2018). The phenotype of *atshi* mutant is similar to that of the gibberellin (GA) biosynthesis deficient mutant, suggesting that *AtSHI* may take part in the GA signaling pathway (Fridborg et al., 1999). Overexpression of the *SHI* gene in *Kalanchoe* and *Poinsettia* results in a compact phenotype (Lutken et al., 2010; Islam et al., 2013). *SHI*, *STY1*, and *STY2* genes can synergistically promote the development of pistil, stamen, and leaf, and their overexpression can also inhibit stem elongation and tapetal dehiscence (Kuusk et al., 2006; Kim et al., 2010). Rice *LRP* is related to plant height through the GA signaling pathway (Duan et al., 2019). *SHI/STYs* are vital for the formation and development of the early lateral root in *Arabidopsis* (Smith and Fedoroff, 1995). Furthermore, *LRP1* can form complexes with *SRS6*, *SHI*, *SRS3*, *SRS7*, and *STY1* and participate in auxin signal transduction and chromatin modification during lateral root development (De Smet et al., 2008; De Rybel et al., 2012). SWIRM domain PAO protein (SWP1) can inhibit LRP1 through histone deacetylation of chromatin. Insertion mutagenesis of *SWP1* or overexpression of *LRP1* can reduce this inhibition and increase root elongation (Krichevsky et al., 2009). In addition, *LRP1* and *STY1* regulate the expression of *YUCCA 4* (*YUC4*) during auxin synthesis (Eklund et al., 2010; Singh et al., 2020). It is reported that *STY1* (*SRS1*) not only regulates auxin biosynthesis and affects the apical pattern of stamen, but also functions in cell proliferation and flowering (Eklund et al., 2010; Staldal et al., 2012).

About 12–20 million years ago, the segregation between *Brassica* species and *Arabidopsis* occurred (Yang et al., 1999; Town et al., 2006). *Brassica* species have achieved triploidization of the whole genome about 5–15 million years ago (Beilstein et al., 2010; Wang et al., 2011). After that, *Brassica oleracea* and *Brassica rapa* were separated about 4.6 million years ago (Liu et al., 2014). *B. napus* (AACC 2*n* = 38) is an allotetraploid, produced by the hybridization of *B. rapa* (AA 2*n* = 20) and *B. oleracea* (CC 2*n* = 18) naturally, about 7,500 years ago (Chalhoub et al., 2014). It has been reported that every *Brassica* species has undergone gene duplication events during evolution. The whole-genome sequencing and assembly had been completed in *B. napus*. 101,040 gene models were generated from 35.5 Gb of sequencing data. The assembled C subgenome (525.8 Mb) was larger than the A subgenome (314.2 Mb; Xie et al., 2020, Yang et al., 2020).

In this study, 195 members of the *SHI/STY* gene family were identified from the 21 land plants, including *P. patens* and *Selaginella moellendorffii*, and a variety of monocotyledons and dicotyledons in the Phytozome database^1^ (Goodstein et al., 2012). Based on the analysis of the molecular evolution characteristics of the *SHI/STY* gene family in terrestrial plants, an evolutionary map was also proposed.

## Materials and methods

### Retrieval of *SHI/STYs* in land plants

Here, all *Arabidopsis* SHI/STY proteins sequences were used to perform a BLAST search in the Phytozome database^2^ (Goodstein et al., 2012) and the Genoscope database for *B. napus* (Chalhoub et al., 2014).^3^ Secondly, the Batch CD-Search^4^ (Marchler-Bauer and Bryant, 2004) was used to screen again the conserved domain of SHI/STYs, and only the sequences with a RING-like zinc-finger domain (DUF702) were selected for further analysis (Fridborg et al., 2001). The physicochemical properties of these SHI/STY proteins were predicted with the ProtParam (Gasteiger et al., 2003). Their subcellular locations were predicted with the CELLO (Chen et al., 2011, 2017).

### Phylogenetic analysis and characterization of *SHI/STYs*

To investigate the evolutionary patterns of these plant *SHI/STYs*, the phylogenetic trees were constructed with the neighbor joining (NJ) method in MEGA7.0.21 (Tamura et al., 2013). The reliability of these trees was evaluated by 1,000 bootstrap replications. Multiple Em for Motif Elicitation (MEME, http://meme-suite.org/tools/meme; Bailey et al., 2006) was used to analyze the conserved motifs of these SHI/STY proteins. The Amazing Optional Gene Viewer in TBtools (Chen et al., 2020) was used to present the distribution of the conserved motifs and the exon-intron structures of SHI/STYs.

### *Cis*-acting element analysis

To analyze the *cis*-acting elements of the *SHI/STY* promoters, TBtools (Chen et al., 2020) was used to obtain the 2,000 bp promoter sequences from the upstream coding sequence (CDS). All the promoters of *SHI/STY* genes in the *Brassica* species were acquired from NCBI.^5^ Three tools, the Gtf/Gff3 Sequence Extractor, the Fasta Extractor, and Amazing Fasta Extractor in TBtools were used to extract the information of promoters. The distribution of *cis*-acting elements in *SHI/STYs* was analyzed with Plantcare^6^ (Lescot et al., 2002).

### Chromosomal location, identification of paralogous *SHI/STY* Genes

The information for the length and location of *SHI/STYs* in the *Brassica* species were obtained from the Phytozome^7^ (Goodstein et al., 2012) and the Genoscope,^8^ respectively. Multiple collinear scanning toolkits (MCScanX) in TBtools (Chen et al., 2020) were used to analyze synteny relationships and gene duplication events among *B. napus*, *A. thaliana*, *B. oleracea*, and *B. rapa*. The *Ks* and *Ka* values of gene pairs were calculated in Tbtools. Duplication time of these gene pairs can be calculated by the frequency (λ) of 1.4 × 10^−8^ homogeneous substitutions per site each year according to *T* = *Ks*/2*λ* (Wang et al., 2011).

### *SHI/STYs* expression analysis in *Brassica napus*

To investigate the expression pattern of *SHI/STYs* in *B. napus*, their RNA-seq data were obtained from the Genoscope database^9^ (Chalhoub et al., 2014). Mev in SOURCEFORGE^10^ was used to analyze their tissue expression levels (Sullivan et al., 2011). In addition, RNA-seq data under the *Sclerotinia sclerotiorum* stress, drought, and heat treatment were obtained from NCBI (GSE169299 and GSE156029), respectively (Clough and Barrett, 2016). These data were standardized based on the log2 scale and clustered and visualized with TBtools.

### Selective pressure analysis

Likelihood-ratio test was used to compare the implemented models. Here, M8 (*ωs* ≥ 1), M8a (*ωs* = 1), M7 (beta), and M5 (gamma) evolution models were used to evaluate selective pressure (Chalhoub et al., 2014). CDS sequences of *SHI/STY* genes were used to calculate selective pressure with Selecton (Chalhoub et al., 2014).^11^ In addition, I-TASSER^12^ (Yang et al., 2015) was used to predict the tertiary structures of BnaA04g04120D, BnaA07g12710D, BnaA03g59180D, and Potri.003G85901.1.p proteins, which were selected as query sequences in different clades in the selective pressure analysis.

### Protein–protein interaction network analysis of SHI/STYs

The protein–protein interaction network of SHI/STYs in *B. napus* was predicted with STRING (version 11.0; https://string-db.org/cgi/input.pl; Szklarczyk et al., 2019). There is no relevant data on *B. napus* in STRING, but *B. rapa* is highly homologous to *B. napus*. Here, all BnSHI/STYs protein sequences were used as the basis of the query to search for the interaction network of homologous proteins in *B. rapas*. The full network type was used to complete the analysis, and the edges represent the physical and functional protein associations. The minimum interaction score required was set to intermediate confidence (0.400).

### Plant material RNA extraction and qRT-PCR

Rape seeds were germinated on Petri dishes in the greenhouse under the conditions of 50 μmol/m2/s light intensity and 70% relative humidity and then grown at 20 ± 5°C, 16 h light /8 h dark. Two-week-old seedlings were treated with Hoagland liquid medium containing plant hormone (50 μM ABA, 10 μM IAA, 75 μM 6-BA) for 24 h. Meanwhile, two-week-old seedlings were treated with cold (4°C), salt (200 mM NaCl) and drought (15%PEG) for 24 h. The whole treated seedlings were then collected and immediately stored at −80°C for RNA extraction. The extraction of total RNA and subsequent synthesis of cDNA were described by Sarwar et al. (Sarwar et al., 2021). *Actin* gene (GenBank ID: XM_013858992) was used as an internal reference. Relative expression level of the *BnSHI/STYs* was measured by the 2^−∆∆Ct^ method. Rape seedlings without any treatment were used as the control. The significant difference between different treatments was measured by *T*-test, and the results were visualized by GraphPad Prism7.4 software (Drafor et al., 2021). All gene-specific primers (Supplementary Table S1) used in this study were designed with BrassicaEDB.^13^

## Results

### Identification and classification of SHI/STY proteins

To identify *SHI/STY* members in land plants, the amino acid sequences of 10 SHI/STYs in *Arabidopsis* were used as queries to perform the BLAST search in the Phytozome database^14^ (Goodstein et al., 2012) and the Genoscope database^15^ (Chalhoub et al., 2014), respectively. As a result, 195 *SHI/STY* genes were identified in 21 land plants. In addition, the DUF702 domain was predicted in all SHI/STY protein sequences by Batch CD-Search^16^ (Marchler-Bauer and Bryant, 2004).

The physical and chemical properties of these identified members were analyzed (Supplementary Table S2). These genes encoded 95–603 amino acids with 9.35 to 61.83 KD and their isoelectric points ranged from 5.56 to 10.12. Subcellular location revealed that 91.8% of SHI/STY proteins were predicted to only locate in the nucleus. In addition, ten members were predicted as nuclear and extracellular localization proteins. This is consistent with the previous report that SHI/STYs are mainly localized in nuclear (Zhang et al., 2015).

**Figure 1:**
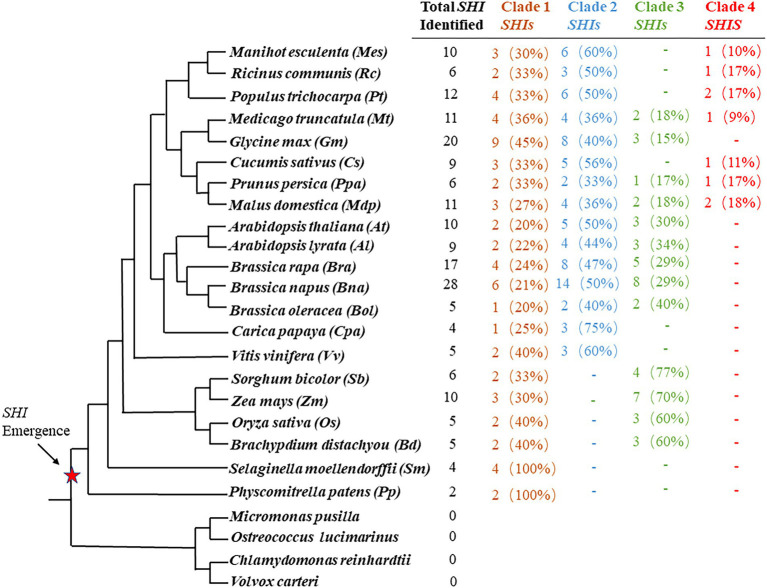
*SHI/STY* genes are identified in land plants. The number of *SHI/STY* genes identified in each sequenced plant genome was listed in sequence. Percentages for each type are listed in parentheses. The red star marks indicate when *SHI/STY* appeared during the evolution of land plants. “-” indicates that there is no *SHI/STY* in this species.

In order to study their evolutionary relationship in land plants, these SHI/STY protein sequences were used to construct phylogenetic trees with the NJ method (Supplementary Figure S1). Combined with the results of conserved motif analysis and phylogenetic tree analysis, SHI/STY proteins in all land plants evolved into four clades (Figure 1; Supplementary Figure S1). Clade1 includes 63 *SHI/STYs* in these 21 land plants. In Clade 1, two and four members existed in *P. patens* and *S. moellendorffii*, respectively, which constitute the earliest *SHI/STY* members of terrestrial plants. Clade 2 is the largest one, which contains 77 members and all of them are dicotyledons. In addition, Clade 3 consists of 46 *SHI/STYs*, all of which come from both monocotyledons and dicotyledons. Clade 4 is a new evolutionary clade. Compared to the other three clades, it only contains 9 *SHI/STY* genes of dicotyledons.

### Motif analyses of SHI/STY proteins in land plants

**Figure 2:**
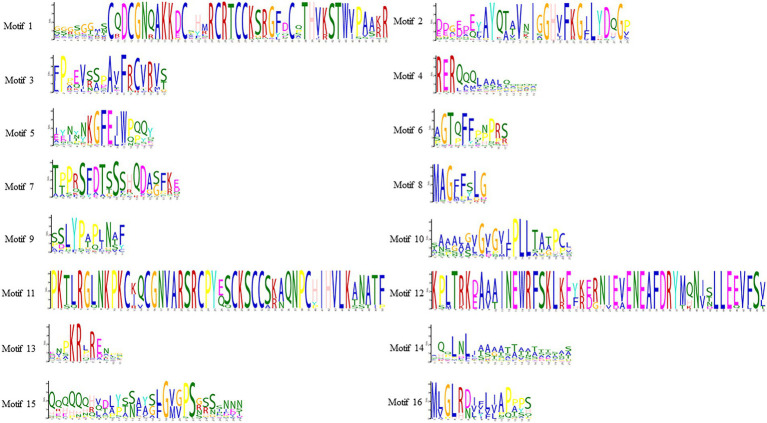
Sequence logos of conserved motifs in SHI/STY proteins. This logo represents the sequence of 16 motifs. The letters represent any amino acid, and the size represents how well it has been preserved at that location. The predicted motif sequence was obtained from the MEME website based on the sequences of SHI/STYs (http://meme-suite.org/tools/meme).

**Figure 3:**
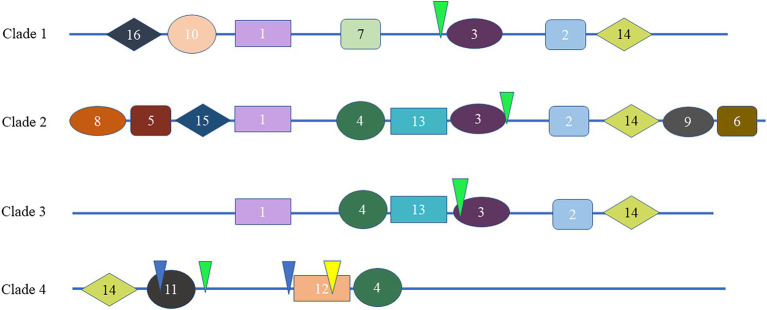
Different motif distribution of the SHI/STY proteins and exon–intron organization in different clades. The different colorful patterning represents the relative positions of motifs on the protein sequence in the different clades. The inverted triangles with yellow, green and blue marks indicate the insertion positions of phase 0, 1 and 2 introns, respectively.

To study the homology domain and conservation degree of the SHI/STY proteins in land plants, MEME was used to perform motif analyses. As a result, 16 conserved motifs were identified (Figure 2; Supplementary Figure S2). The motif distribution of SHI/STY proteins was similar in the same clade. The RING-like zinc finger domain (Motif 1) and the IGGH domain (Motif 2) are conserved in most of the SHI/STYs. Besides, Motif 14 and Motif 4 are conserved in SHI/STYs. In addition, different motif distribution is found (Figure 3). For example, Motif 10 and Motif 16 are only identified in Clade 1, and Motif 8 and Motif 15 are conserved in Clade 2. The number of motifs is the highest in Clade 2 and the lowest in Clade 4. It can be predicted that the SHI/STY proteins might lost or obtain some motifs in the long process of genetic evolution.

To understand the diversity of SHI/STYs, the gene organization and intron phase distribution were analyzed (Figure 3). Genes with the same phylogenetic group usually have similar exon-intron structures. Statistical analysis showed that only one intron was conserved in Clades 1, 2, and 3, while four conserved introns were found in Clade 4. All eukaryotes have phase 0, phase 1 and phase 2 introns, among which phase 0 introns account for the highest proportion and have the highest conservation (Long et al., 1995). It is clear that the phase 1 introns were conserved in most *SHI/STYs*, while introns in Clade 4 are different from the others. The conserved introns in Clades 1–3 suggest that the *SHI/STY* gene family is relatively conserved during the evolution of land plants. However, the new intron pattern in Clade 4 reflects the structure change of *SHI/STY* genes during the evolution from lower plants to higher ones.

### Chromosomal distribution and duplication of *SHI/STY* genes

**Figure 4:**
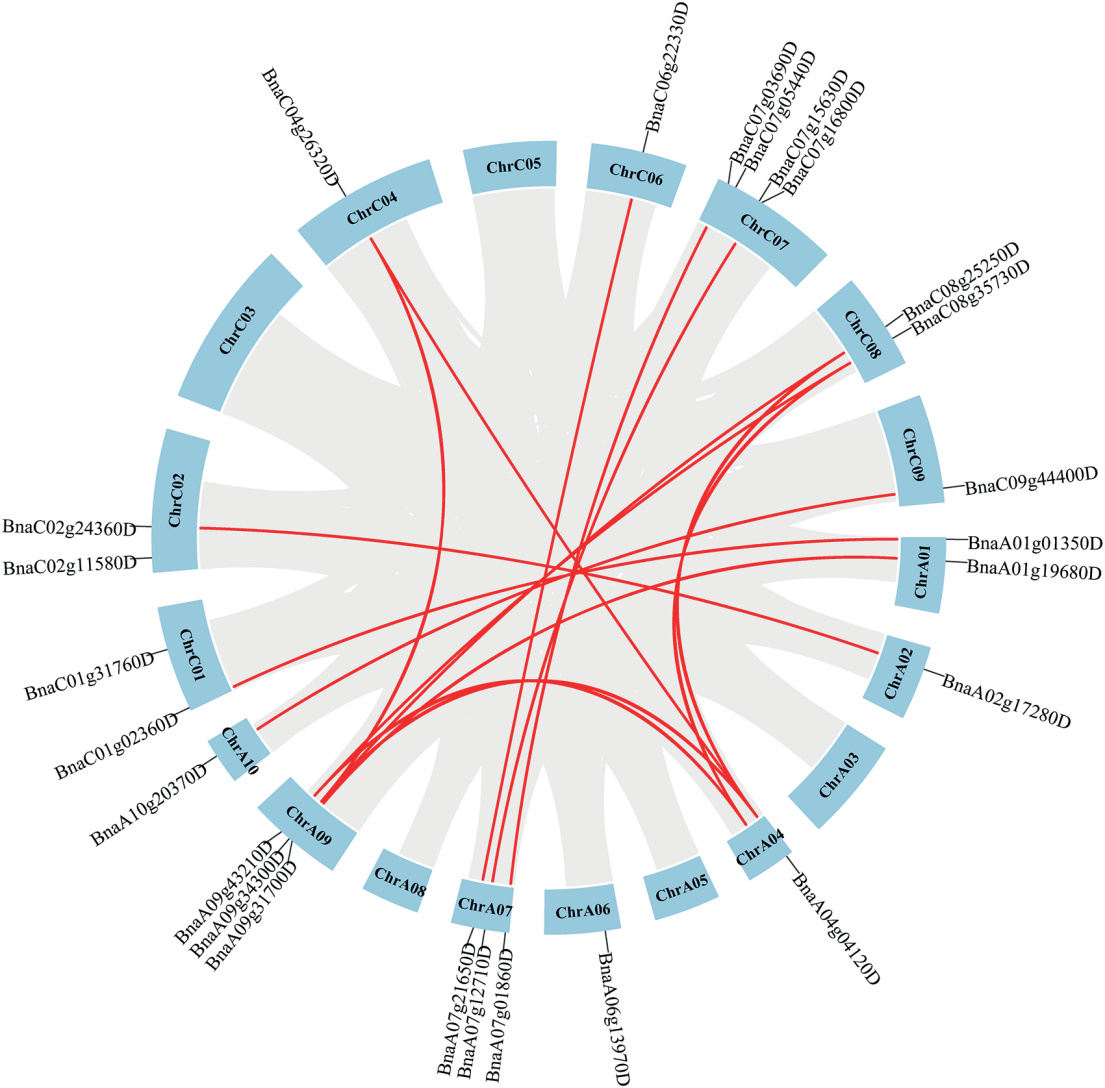
Chromosome distribution of *BnSHI/STYs*. 25 *BnSHI/STYs* are distributed on 19 chromosomes of *Brassica napus*. In addition, 3 *BnSHI/STYs* are not mapped to chromosomes. The duplicated *BnSHI/STY* gene pairs were indicated in red lines. The number of chromosomes is displayed on each chromosome.

**Figure 5:**
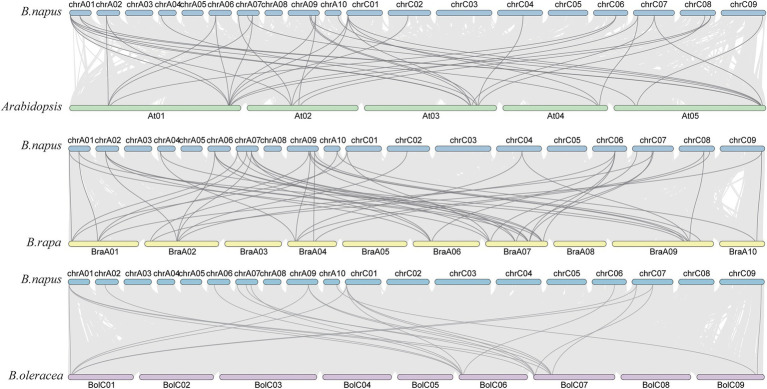
The collinear relationship of *SHI/STYs* on the chromosome. A chromosome is represented by a bar. The green, yellow, and purple bars represent the chromosomes of *Arabidopsis*, *B. rapa*, and *B. oleracea*, respectively. The collinear blocks (between the genomes of *B. napus* and *Arabidopsis*, *B. napus* and *B. rapa*, *B. napus* and *B. oleracea*) were indicated in the gray lines.

It was reported that duplication occurred in both A and C subgenomes of *B. napus* (Chalhoub et al., 2014; Panchy et al., 2016). To analyze duplication events of *SHI/STYs*, MCScanX of TBtools was used to investigate their duplication events in the *Brassica* species (Figures 4, 5). *BnSHI/STYs* were first located onto the chromosomes of *B. napus*. The result shows that 13 *BnSHI/STYs* were located on C chromosomes, while 12 *BnSHI/STYs* were located on A chromosomes. Chromosome C07 contained the largest number of *BnSHI/STY* genes (four genes), but no *BnSHI/STY* was identified on Chromosomes C03, C05, A03, A05 and A08.

Next, syntenic relationships of the *SHI/STYs* between *B. napus* and other three species (*B. rapa*, *B. oleracea*, and *Arabidopsis*) were analyzed to investigate their evolutionary relationship. Their location maps are shown in Figure 5. Collinearity analysis revealed that *B. napus* had more orthologous *SHI/STYs* than *Arabidopsis*, *B. rapa*, and *B. oleracea*. *B. rapa* has the most syntenic *SHI/STY* genes with *B. napus*, followed by *Arabidopsis*. *B. oleracea* has the least syntenic *SHI/STY* genes with *B. napus.* Next, we calculated the divergence time of duplicated gene pairs in these four species (Supplementary Table S3). Among these *SHI/STYs*, 20 pairs of duplicate genes were identified, including 3 pairs in *Arabidopsis*, 11 pairs in *B. napus*, 5 pairs in *B. olerace* and 1 pair in *B. rapa*. *SHI/STYs* duplicated earliest at 113 million years ago in *Arabidopsis*, and followed by *B. rapa* and *B. napus* about 54 and 27 million years ago. In *B. napus*, 81.8% of duplicated gene pairs were caused by WGD. These results indicated that gene duplication promoted the amplification of *SHI/STY* genes in *B. napus* genome to a large extent, and WGD or segmental duplication was the main driver.

### Analysis of the *cis*-acting element of *SHI/STYs*

**Figure 6:**
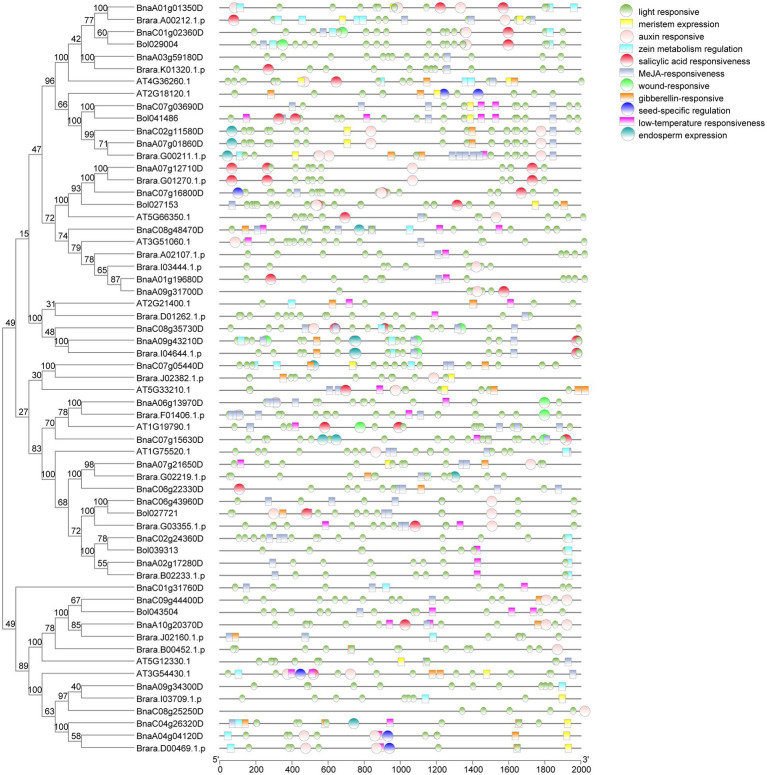
*Cis*-element analysis in the promoter regions of the *SHI/STY* genes. The PlantCARE software was used to determine the presence of different *c*is-acting elements. The different *cis*-elements were represented in different colored boxes.

To identify the functional differences of *SHI/STYs* in four *Brassica* species, we analyzed the *cis-*elements in the promoters of *SHI/STY* genes with PlantCARE software (Figure 6). three kinds of *cis*-acting elements were found in the promoter region of the *SHI/STY* gene family. Most *cis*-elements of *BnSHI/STYs* promoters are involved in plant growth and development, hormonal responsive and abiotic stress responsive. Some *cis*-elements, like meristem expression, endosperm expression, seed-specific regulation and light responsive are the main *cis*-elements involved in plant growth and development regulation. Light responsive related elements are most common in the *SHI/STY* promoters, indicating that the *SHI/STY* gene family could be induced by light to regulate plant growth and development. Low-temperature responsiveness and wound responsive are the main members of the regulation of abiotic stress. At the same time, we also found that elements related to hormone response, including salicylic acid responsiveness, MeJA-responsiveness, and gibberellin-responsiveness, accounted for a large proportion. These results suggest that *SHI/STYs* not only regulate plant growth and development, but also play an important role in hormone response and abiotic stress. However, an element related to endosperm expression was found only in the promoters of *B. napus* and *B. rapas*. MeJA-related responsiveness and salicylic acid (SA) responsiveness were identified mainly in the second category. We found that the number of MeJA responsiveness elements was the largest, followed by the SA responsiveness elements. In addition, some *cis*-acting elements related to abiotic stresse responses were identified, such as wound-responsive and low-temperature responsiveness.

Differences of the *cis*-elements in the promoter region between homologous genes may appear during the process of gene duplication, and the difference in *cis*-element might lead to different expressions of duplicated genes and increase the divergence of gene function. For example, there are more low-temperature responsiveness related elements in duplicated gene *BnaA10g20370D* than in *BnaC09g44400D* (Figure 6), and it could be found that the former were up-regulated when *B. napus* suffered cold. The *cis-*elements in the duplicated gene (*BnaC07g12710D*/*BnaA06g13970D*) were different. These two genes are expressed in different tissues, and *BnaC07g12710D* is highly expressed in the stem, but the other is expressed in the bud. Therefore, the difference in *SHI/STY* gene expression is caused by the difference in *cis-*elements.

### Positive selection in *SHI/STY* gene family

**Figure 7:**
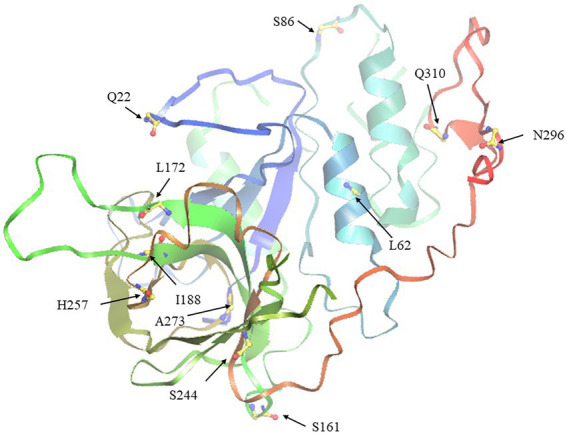
The tertiary spatial structure of BnSHI/STY protein in Clade 3. The eleven predicted positive selection sites were marked with arrows.

To describe the selective pressure on different clades during the evolutionary process, the *Ka/Ks* values were calculated. As a result, the *Ka/Ks* values of the four clades were all less than 0.5, suggesting that the *SHI/STY* gene family could have experienced intense purifying selection in the long evolutionary process (Table 1). The *Ka/Ks* value of Clade 3 is the largest, ranging from 0.45 to 0.49, while the *Ka/Ks* value of Clade 1 is the smallest, ranging from 0.25 to 0.27. It indicates that the purification effect of Clade 1 is stronger than others. Here, four models (M7, M8, M8a, and M5) were used to calculate their *Ka/Ks* values. And only M8 and M5 models identified some positive selection sites in Clade 1, Clade 3 and Clade 4. Next, the tertiary structure of SHI/STY proteins was predicted to locate the positive selection sites (Table 1; Figure 7). The results showed that most of the positive selection sites were located in Motif 1 and Motif 2. These two motifs code the highly conserved IGGH domain and RING-like zinc finger domain, respectively. Its ability to bind to RNA, protein, and lipid substrates demonstrates that it functions as a transcriptional activator (Fridborg et al., 2001; Kuusk et al., 2006; Zhao et al., 2020). Amino acids in these two motifs are involved in maintaining the structure and functions of the motifs. The changes in these amino acids may have a significant effect on the functions of SHI/STY proteins.

**Table 1:** Likelihood values and parameter estimates of positive selection in codons of *SHI/STY* gene family in 21 land plants.

Gene branches	Selection model	*Ka/Ks*	Log-likelihood	Positive-selection sites
Clade 1	M8 (ωs ≥ 1)	0.268208955	−36522.3	–
	M8a (ωs = 1)	0.258084577	−36,523	–
	M7 (beta)	0.264059701	−36551.2	–
	M5 (gamma)	0.273109453	−36563.1	A29/H84/G125
Clade 2	M8 (ωs ≥ 1)	0.309850746	−37365.8	–
	M8a (ωs = 1)	0.309537313	−37368.6	–
	M7 (beta)	0.302023881	−37,363	–
	M5 (gamma)	0.32722597	−37445.8	–
Clade 3	M8 (ωs ≥ 1)	0.460069841	−17826.9	–
	M8a (ωs = 1)	0.458142857	−17820.1	–
	M7 (beta)	0.466453968	−17823.2	–
	M5 (gamma)	0.493180952	−17849.6	Q22/S86/I88/T170/L172/S244/M247/G250/H257/A273/Q310/L62/T111/S161/T171/M189/G252/G253/D274/S295/N296
Clade 4	M8 (ωs ≥ 1)	0.390965147	−7075.45	G26/V333/T15/V28/S31/E195/D202/A204/L250/L252/S256/V257/N259/Q260/V263/A270/A305/R313/T382/Q336
	M8a (ωs = 1)	0.383171582	−7077.51	–
	M7 (beta)	0.355691689	−7079.97	–
	M5 (gamma)	0.393772118	−7076.06	G26/V333/T15/E195/D202/A204/L250/L252/S256/V257/N259/Q260/A270/A305/R313/T328/Q336/N11/Q17/V28/S31/L188/S199/V263/P267/S272/Q310/S314/L324/S359

### The predicted protein–protein interaction network of SHI/STYs

**Figure 8:**
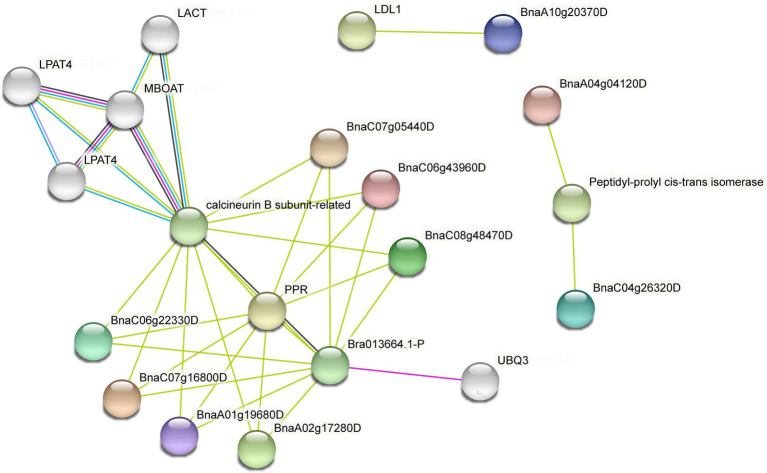
The protein–protein interaction network of SHI/STYs. Eleven SHI/STYs were involved in the construction of the protein–protein interaction network. Fifty-three protein–protein interactions were found between SHI/STYs and some other proteins. The colorful balls represent different proteins and the line between proteins indicates the interaction between the two proteins.

Protein–protein interaction networks are a useful and convenient tool for the systematic study of complex biological activities in cells (Hao et al., 2016). To fully understand the function of SHI/STYs in *B. napus*, the interaction network of SHI/STYs was predicted using the STRING (Figure 8). The result indicated that the SHI/STYs interacted with many different proteins like lysophosphatidic acid acyltransferase (LPAT4), membrane-bound O-acyltransferases (MBOATs), Pentatricopeptide repeat (PPR), ubiquitins (UBQ3), low-density lipoprotein (LDL), calcineurin B subunit-related (CnB) and peptidyl-prolyl cis-trans isomerase.

LPAT is crucial to the biosynthesis of phospholipids and TAG due to its strong flux control of the *de novo* phospholipid biosynthesis pathway (Angkawijaya et al., 2019). In *Arabidopsis*, LPAT4 plays a role in the N starvation response by playing a function in endoplasmic-reticulum-localized denovo of glycerolipid biosynthesis (Angkawijaya et al., 2019). MBOAT is a large family of integral transmembrane enzymes that function in lipid biosynthesis or phospholipid remodeling and cell surface protection (Hofmann, 2000; Chang et al., 2006; Chang and Magee, 2009; Liu et al., 2012). The result indicated that SHI/STYs in *B. napus* interact indirectly with these two proteins. Thus, SHI/STYs might be involved in phospholipid and TAG biosynthesis, lipid synthesis, phospholipid remodeling, and cell surface protection. The CnB can regulate the proteasome pathway (Li et al., 2011). The plant PPR proteins may be an evolutionary adaption to complicated RNA metabolism in mitochondria and chloroplasts (Germain et al., 2013). Some interaction networks between proteins (CnB, PPR) and BnSHI/STYs (BnaC06g22330D, BnaC07g05440D, etc.) suggested that BnSHI/STYs might be involved in the proteasome pathway and RNA metabolism. Furthermore, the expression pattern of UBQ3 significantly changed when subjected to cold stress (Ding et al., 2020), suggesting that UBQ3 is associated with cold stress. It can be speculated that these BnSHI/STYs interacting with UBQ3 may also be involved in the cold stress response. Peptidyl-prolyl cis-trans isomerase can regulate the phosphorylation of protein and plays role in cell proliferation and transformation (Chen et al., 2018). In our protein interaction network, BnaA04g04120D and BnaC04g26320D interact with this protein, which means that they may also play homologous functions in cell proliferation and transformation. Above all, by influencing regulatory pathways and biological processes, the SHI/STY proteins are crucial for plant growth and development.

### Analysis of the expression patterns of *Brassica SHI/STY* genes

**Figure 9:**
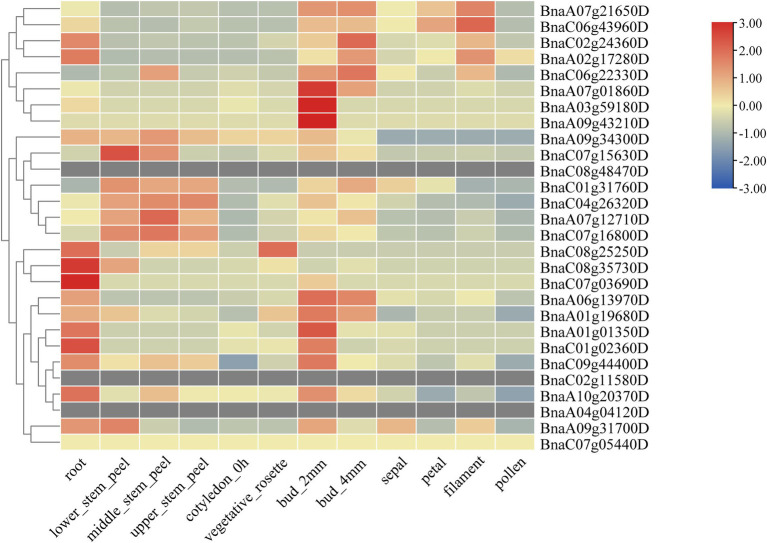
The expression patterns of *BnSHI/STYs* in different tissues. *BnSHI/STYs* are clustered by hierarchical clustering. The different color box represents expression quantity in different tissues, red to blue means expression lever from high to lower.

To fully study the expression patterns of *BnSHI/STYs* in different tissues and stress conditions, TBtools was used to analyze their expression levels with the obtained RNA-seq data from the NCBI database (Figure 9). The expression pattern of *BnSHI/STYs* can be roughly divided into three types according to their expression levels. The first type is the majority, such as *BnaC08g35730D*, *BnaA06g13970D*, and *BnaC01g02360D*, which are mainly expressed in roots and buds, and some of them are expressed in the stem. The second type is mainly expressed in the stem, such as *BnaC04g26320D*, *BnaC07g16800D*, and *BnaA07g12710D*, etc. The *BnSHI/STYs* in the third type are mainly expressed in buds, filaments, and roots, such as *BnaA07g21650D*, *BnaC06g43960D*, and *BnaC02g24360D*, etc. Some of them are also expressed in stems and petals such as *BnaC06g22330D*. Combined with the expression levels, *BnSHI/STYs* within the first type mainly belong to Clades 1 and 3, indicating that they are mainly involved in root growth and development. The second type was mainly distributed in Clade 2 and was related to the growth and development of the stem.

**Figure 10:**
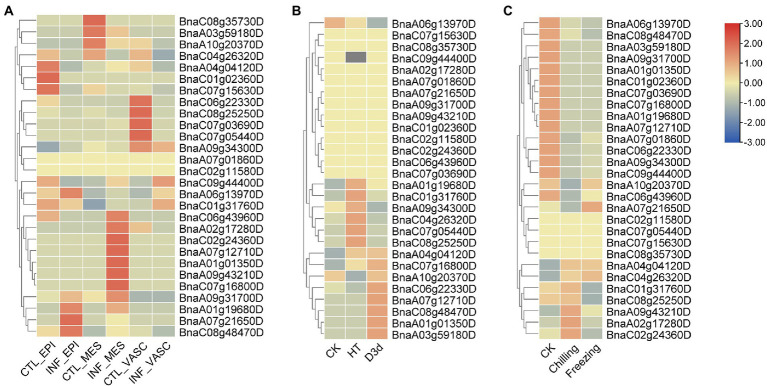
The expression patterns of *BnSHI/STYs*. **(A)** The expression pattern of *BnSHI/STYs* after infection by *Sclerotinia sclerotiorum*. **(B)** The expression pattern of *BnSHI/STY* in *B. napus* under abiotic stress. **(C)** The expression pattern of *BnSHI/STYs* in cold stress. RNA sequence data that were treated with cold shock at temperatures of chill (4°C) and freezing (4°C) were used to complete this figure, and “CK” was treated at 25°C. Genes with high expression levels are shown in red, and genes with low expression levels are shown in blue. The gray box indicates that the expression data of this gene were not found in the database.

Next, the expression patterns of *BnSHI/STYs* infected by *S. sclerotiorum*, and under drought, cold and heat stress were also analyzed. 5, 10 and 3 *BnSHI/STYs* were up-regulated in the epidermis, mesophyll, and vascular, respectively, compared with control (Figure 10A). Moreover, 7 *BnSHI/STYs* were up-regulated under high temperature, and another 7 *BnSHI/STYs* were up-regulated when *B. napus* suffered drought treatment (Figures 10B,C). Some *SHI/STY* genes such as *BnaC08g48470D*, *BnaA01g19680D* and *BnaC07g16800D*, etc., were up-regulated under both high-temperature and drought. This result indicates that these *BnSHI/STYs* may play important functions when plants suffered biological and abiotic stress. We also found that about 53.6% of *SHI/STYs* were down-regulated under cold stress, such as *BnaA01g19680D*, *BnaA06g13970D* and *BnaC08g48470D*, etc. And most of the down-regulated members belong to the Clade 3, and up-regulated *SHI/STYs* belong to the Clades 1 and 2, suggesting that *SHI/STYs* in Clades 1 and 2 might help plants to respond to cold stress.

The expression patterns of duplicated genes were also investigated under different stress treatments. For example, *BnaA07g21650D* was up-regulated under cold and infected by *S. sclerotiorum*, but was down-regulated under drought. While its duplicated gene (*BnaC06g22330D*) presented the opposite expression pattern. This indicates that the functions of duplicated genes have diverged during evolution.

### Expression patterns of *BnSHI/STY* genes under different stress and hormone treatments

**Figure 11:**
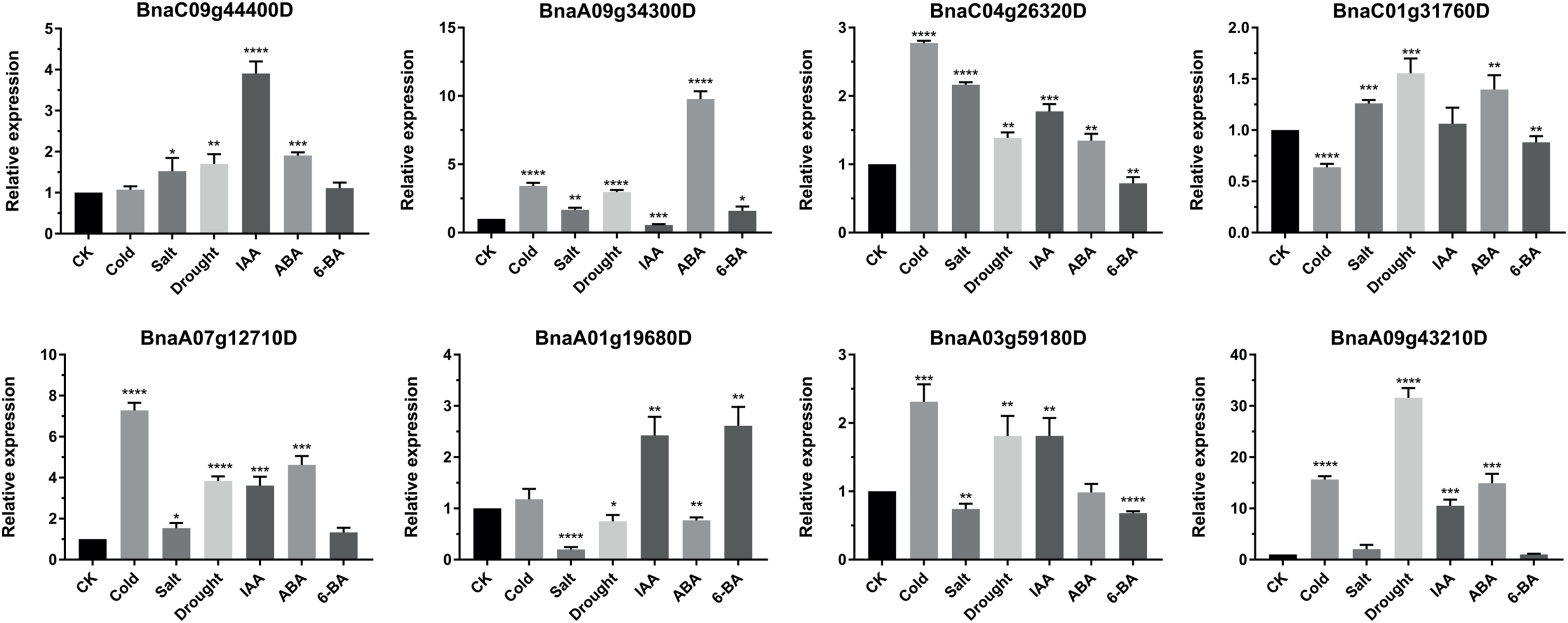
Expression levels of eight *BnSHI/STYs* under different abiotic stresses and hormone treatments. The selected *BnSHI/STYs* were normalized concerning the reference gene (Actin). The *X*-axis corresponds to different abiotic stress treatments and hormone treatments. The values on the *Y*-axis represent the mean ± SD of three biological replicates. Asterias on vertical bar shows significant difference at ^*^p < 0.05, ^**^p < 0.01, ^***^p < 0.001, ^****^p < 0.0001.

To further explore and understand the possible functions of *BnSHI/STYs* under biotic and abiotic stresses, eight *BnSHI/STYs* were selected randomly and their expression levels in rape seedlings treated with different abiotic stresses and hormones were measured by qRT-PCR (Figure 11). Overall, the expression pattern of *BnSHI/STYs* was different under different stress treatments. For example, *BnaC09g44400D* was significantly upregulated under all stresses except cold and 6-BA. The expression level of *BnaA09g34300D* was up-regulated under various stresses, but significantly down-regulated under IAA treatment. *BnaC04g26320D* was significantly upregulated in all stresses except 6-BA. Under abiotic stress, 5 *BnSHI/STY* genes were upregulated under cold stress, seven genes were upregulated under drought stress, while five genes were upregulated under salt stress. Compared with CK, salt stress had the least effect on the expression level of *BnSHI/STYs* selected, and the expression level of *BnaA09g34300D* was up-regulated by about 1.5 times. *BnaA09g43210D* was most affected by abiotic stress, and the expression level of *BnaA09g43210D* increased about 16 times under cold stress, and about 31 times under drought stress. In the experiment of IAA, ABA, 6-BA hormone treatment, the expression level of *BnSHI/STYs* in six selected samples was up-regulated by IAA, and the expression level of *BnaA09g34300D* was down-regulated. Six selected *BnSHI/STYs* were up-regulated by ABA and *BnaA01g19680D* down-regulated. The expression pattern of *BnaA09g43210D* was most up-regulated by IAA and ABA, which was about 11 and 15 times that of CK. The expression levels of 4 selected *BnSHI/STYs* were up-regulated by 6-BA, but no *BnSHI/STY* was down-regulated by 6-BA. As a result, our data further suggest that *SHI/STYs* are involved in plant stress and hormone responsive.

## Discussion

**Figure 12:**
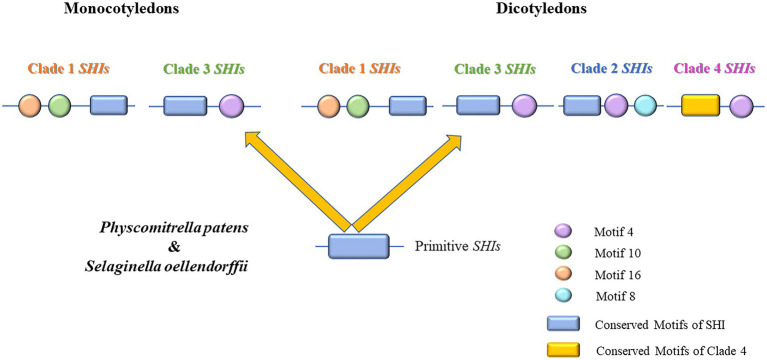
Evolution scenario of the *SHI/STY* gene family in land plants. In *Physcomitrella patens* and *Selaginella oellendorffii*, the *SHI/STY* gene family emerged. The *SHI/STY* gene family is formed by adding different motifs at both ends of conserved domains to form Clade 1 and Clade 3 in monocotyledons. In dicotyledons, the Clade 3 gene was further duplicated and gradually evolved into three branches through loss or acquisition of new motifs (some retained the characteristics of Clade 3, some acquired motif 8 and evolved into Clade 2, while the conserved motif in Clade 4 was lost).

In *Arabidopsis*, the *SHI/STY* gene family has been extensively studied in recent years (Singh et al., 2020). The *SHI/STY* protein contains a zinc finger-like RING domain that is conserved at the N-terminal (Fridborg et al., 2001). Members of *SHI/STY* gene family were important for the plant by playing functions in the formation and development of lateral roots (Smith and Fedoroff, 1995). The function of *SHI/STY* has been reported in some plants as well, for example, by binding to *cis*-element in YUCCA4 gene promoter directly, *AtLRP1* has transcriptional activity in auxin biosynthesis (Singh et al., 2020). Consequently, the *SHI/STY* genes play different functions in the coordination of multiple hormonal pathways that regulate growth and development (Yang et al., 2020). Therefore, the study of the evolution of the *SHI/STY* gene family is of great significance. In the aggregate, 195 *SHI/STY* genes were identified in 21 land plants, and divided into 4 clades (Figure 1). *SHI/STYs* in Clade 1 appear earliest and have been identified in *P. patens* and *S. moellendorffii*. *SHI/STY* genes in monocotyledons are only present in Clade 1 and Clade 3. The members of Clade 2 were identified from dicotyledons, and the members of Clade 4 were identified only from higher dicotyledons. Based on the distribution of *SHI/STY* members and their conserved motifs in different clades, a model was speculated to describe the evolution scenario of the *SHI/STY* gene family in land plants (Figure 12). The first *SHI/STYs* containing conserved Motif 1 appeared in *P. patrella* and *S. moellendorffii*. Among monocotyledons, some *SHI/STYs* obtained Motif 10 and Motif 16 and were conserved in dicotyledons, forming Clade 1. Another group of *SHI/STYs* acquired Motif 4, and these *SHI/STYs* evolved in three different directions. Members of dicotyledons and monocotyledons with similar motif structures are divided into Clade 3, some *SHI/STY* acquired Motif 8 in the evolution process, and this kind of *SHI/STY* is divided into Clade 2. Clade 4 evolved from Clade 3, and in the evolution process, conserved Motif 1 was lost in *SHI/STYs* in Clade 4, but a new conserved motif was obtained. There are more *SHI/STYs* in *B. napus* than in other species. The gene structure and distribution pattern of conserved motifs are consistent with the position of genes in the phylogenetic tree. Hence, SHI/STY proteins might not be conserved between the species, but are more likely to have experienced gene duplication events to expand their numbers in land plants.

The zinc finger domain similar to RING (CX2CX7CX4CX2C2X6C, Motif 1) is a type of RING domain (C3H2C3 or C3HC4), which was identified as a DNA binding motif (Kaulen et al., 1991). This domain can play an important role in many physiological and biochemical processes by binding to RNA, protein, and lipid substrates (Berg and Shi, 1996; Elenbaas et al., 1996). The IGGH domain (motif 2) is positioned in the C-terminal and contains four highly conserved residues in the region, which plays the function of a transcriptional activator (Fridborg et al., 2001). In addition, these results suggest that *SHI/STYs* may play an important role in regulating gene transcription. Within each clade, the variety and number of conserved motifs are similar. As a result, it can be found that the difference in motifs between clades might cause the divergence of gene function in the *SHI/STY* gene family.

Previous research suggests that intron-rich genes might be ancestral and that intron-poor genes might be caused by intron losing (Roy, 2006). Genes with more introns provide variant proteins that may play different roles in biological processes by providing more opportunities for alternative splicing (Min et al., 2015). In *Arabidopsis* and rice, genes with fewer introns are more likely to involve in resistance to salt stress and drought response than gene families with more introns (Liu et al., 2021). Our results show that Clade 4 has more introns than the other three branches, and Clade 4 is relatively new. Naturally, it could be predicted that *SHI/STYs* in Clade 4 might obtain introns to adapt to the environmental change in the long evolutionary process.

Gene duplication is quite common and crucial for all species and plays an important role in species evolution (Lynch and Conery, 2000). It promotes the diversity of gene families greatly, and increases the complexity and the size of the genome (Kong et al., 2007; Panchy et al., 2016). Tandem duplication WGD are two main mechanisms of gene duplication (Zhu et al., 2014). Since most plants have undergone polyploidization events during their evolution, and WGD and segmental duplication are prevalent in plant genomes (Cannon et al., 2004; Parkin et al., 2005). Previous studies have shown that *Brassica* plants have experienced extensive gene duplication events during evolution (Cheng et al., 2013; Chalhoub et al., 2014). *B. napus* is an allopolyploid production which produced naturally by the hybridization of *B. rapa* and *B. oleracea* (Chalhoub et al., 2014). About 13 million years ago the *Brassica* genome was tripled (Yang et al., 1999; Lysak et al., 2005). Studies have revealed that *Brassica* species like *B. oleracea* and *B. rapa* diverged from *A. thaliana* during a triplication of the whole genome between 20 and 40 million years ago (Cheng et al., 2013; Chalhoub et al., 2014; Dun et al., 2014). In the diploid ancestors of *B. napus*, the divergence time of the A and C genome segments spanned about 0.12–1.37 million years ago (Cheung et al., 2009). The *SHI/STY* genes might undergo duplication events during the evolutionary process because of their similar motif structure and exon-intron pattern in each subgroup. The common mutation rate (1.4 × 10^−8^ synonym substitutions per site per year) was used to estimate the time of *BnSHI/STYs* divergence (Wang et al., 2011). It was found that most of all *BnSHI/STYs* were caused by gene duplication events (Supplementary Table S4). 20 *BnSHI/STYs* of them were derived from WGD or segmental duplications, and the rest of the 8 *BnSHI/STY* genes were caused by dispersed duplications. All *BnSHI/STYs* had been duplicated before the divergence of the A and C genomes, except three gene pairs (*BnaA07g01860D/BnaC02g11580D*, *BnaA07g21650D/BnaC06g22330D*, and *BnaC02g24360D/BnaA02g17280D*; Supplementary Table S4). The ratio of the non-synonymous substitution rate to the synonymous substitution rate, namely *Ka/Ks*, is an important parameter to analyze sequence evolution (Sueoka, 1992; Hurst, 2002). *Ka/Ks* > 1 is potential evidence of positive selection during gene differentiation, while *Ka/Ks* < 1 usually denotes negative selection/purified selection (Guo et al., 2017). Our study found that *Ka/Ks* ratios of all clades were less than 1, suggesting that purification selection plays a role after *SHI/STYs* differentiation. Most of the *SHI/STYs* duplicated after triploids of the whole genome but before the divergence of A and C genomes. The result of collinearity analysis indicated that there are many homologous *SHI/STYs* between *B. napus* and *Arabidopsis*, *B. napus* and *B. rapa*, *B. napus* and *B. oleracea*, respectively. 71.4 percent of *BnSHI/STYs* are generated by the WGD. Therefore, WGD is the main driver of S*HI/STYs* expansion in the genome of *B. napus*.

*Cis-*acting elements in promoters play crucial roles in regulating gene expression by binding to transcription factors (Maire et al., 1989; Otsuki and Yamamoto, 2020). The *cis*-acting elements in the promoter regions of genes are closely related to the expression pattern of genes (Chow et al., 2018). In addition, the same regulatory elements in gene promoters suggest that they may have similar regulation functions (Xie et al., 2020). Light responsive related elements are most common in all *SHI/STYs* promoters, indicating that light signals may be involved in S*HI/STYs* regulating the growth and development of the *Brassica* species. There are no same *cis*-acting elements in duplicated genes. Most of the duplicated genes in these four *Brassica* species have different *cis*-acting elements pattern. For example, *Bol027721* contains a zein metabolism regulation element, which does not exist in its duplicated gene *Bol039313*. Low-temperature responsiveness-related element exists in most *SHI/STYs* except *BnaA03g59180D*, *BnaC07g05440D*. It suggests that not all *cis*-acting elements were conserved in each promoter of duplicated *SHI/STYs*. The variety of *cis*-acting elements in the promoters, indicates that these *SHI/STYs* might be associated with abundant regulatory pathways and biological processes. The diversity of *cis*-acting elements in duplicated genes indicates that the responses of genes to biotic and abiotic stresses have changed, which is the molecular basis of gene functional diversity. The results suggest that *SHI/STY* could play an important role in regulating hormone responses and responding to abiotic stresses to impact growth and development in *Brassica* species.

Abiotic stress changes plant physiological processes by affecting gene expression, RNA or protein stability, and ion transport (Kosova et al., 2015). Temperature and drought are two serious factors that jeopardize plant growth and development. Over time, plants have evolved many survival mechanisms to respond to stress, such as developing specific morphological characteristics and regulating metabolic pathways (Vijayaraghavareddy et al., 2020). The first plant organ to sense salt stress is the root, which immediately transmits this signal to the leaves through the stem, leading to stomatal closure and reducing water loss (Cosgrove and Hedrich, 1991; Christmann et al., 2013). Previous research has shown that *SHI/STYs* play a crucial role in the growth, development, and responses to biotic and abiotic stress of plants (Yang et al., 2020). Plant hormones are necessary for plant growth and development and play an important role in stress response (Shu et al., 2016). It can find that most of all *BnSHI/STYs* were highly expressed in root and bud, and most of them were up-regulated under drought stress, such as *BnaC03g59180D*, *BnaA01g01350D*, *BnaC08g48470D*, et al. Some *SHI/STYs* such as *BnaC01g31760D*, *BnaA07g12710D*, *BnaC07g16800D* were expressed in the stem. Duplicated gene pairs (*BnaA06g13970D/BnaC01g31760D*) have different expression patterns (Figure 10).

When the plant suffered an infection of *S. sclerotiorum*., some *SHI/STYs* were down-regulated. Some members of them, such as *BnaA03g59180D*, *BnaA10g20370D*, *BnaA04g04120D* and *BnaC06g22330D*, were up-regulated under drought, while *BnaC04g26320D*, *BnaC08g25250D*, *BnaC07g05440D* and *BnaA09g34300D* were up-regulated under high-temperature. Furthermore, the expression level of the *SHI/STY* genes in Clade 1 was higher than in other clades, indicating that the *SHI/STY* genes in Clade 1 could be more crucial for the growth and development of *Brassica*. Differences in expression patterns of these genes may be caused by changes in the upstream *cis*-acting elements of allopolyploid genes formed by natural polyploids and their adaptation to the environment. As a result, it can be summarized that *BnSHI/STYs* can not only influence the growth and development of *B. napus* but can help the *B. napus* to adapt to biological and abiotic stress such as cold, heat, and drought. The qRT-PCR data further showed that *BnSHI/STYs* expression levels were affected by different abiotic stresses and different hormone treatments (Figure 11). Under cold and drought stress, the expression lever of some *SHI/STYs* was raised, such as *BnaA07g12710D*, *BnaA03g59180D*, and *BnaA09G43210D*, which is consistent with the results of Figure 10B. Under the treatment of plant hormones, the expression of most *SHI/STYs* was up-regulated. The results of homeopathic element analysis showed that these *SHI/STYs* have low-temperature responsiveness and Auxin responsiveness. These results suggest that members of the *SHI/STY* gene family are involved in plant response to external stress.

## Conclusion

In this paper, 195 *SHI/STY* genes were identified in 21 land plants. According to phylogenetic analysis, gene structure, and motif distribution, these genes were further divided into four clades. The difference in motifs between clades might cause the divergence of gene function in the *SHI/STY* gene family. Collinearity analysis of four *Brassica* shows that the *SHI/STY* gene family is relatively conserved in the evolution of *B. rapa*, *Arabidopsis*, *B. oleracea,* and *B. napus*. WGD is the main factor of *SHI/STY*s expansion. The *SHI/STY* genes might have occurred intense purifying selection in the evolutionary process. The different types of *cis*-element in most members of *SHI/STY* gene family indicate that they might be associated with abundant regulatory pathways and biological processes. This is consistent with the result of the protein–protein interaction network analysis. The expression analysis indicated that *SHI/STYs* were expressed in different tissues and regulated by various abiotic and biological stresses. The qRT-PCR data further suggest that *SHI/STYs* are involved in plant stress and hormone response and play an important role in plant growth and development. Finally, an evolutionary model was proposed based on the evolution of *SHI/STY* gene family.

## Data availability statement

The datasets presented in this study can be found in online repositories. The names of the repository/repositories and accession number(s) can be found in the article/Supplementary material.

## Author contributions

DF is the executor of the study, mainly completing the basic analysis of data and writing the first draft of the paper. WZ, XC, FH, and ZY participated in the analysis of the results. JC is the project leader, directing the research design and final revision of data analysis results, as well as writing and revising the paper. All authors contributed to the article and approved the submitted version.

## Funding

This research was supported by grants from the National Science Foundation of China (No. 31871655 and 32072019).

## Conflict of interest

The authors declare that the research was conducted in the absence of any commercial or financial relationships that could be construed as a potential conflict of interest.

## Publisher’s note

All claims expressed in this article are solely those of the authors and do not necessarily represent those of their affiliated organizations, or those of the publisher, the editors and the reviewers. Any product that may be evaluated in this article, or claim that may be made by its manufacturer, is not guaranteed or endorsed by the publisher.

## Footnotes

^1^ https://phytozome.jgi.doe.gov/pz/portal

^2^ https://phytozome.jgi.doe.gov/pz/portal

^3^ http://www.genoscope.cns.fr/brassicanapus

^4^ https://www.ncbi.nlm.nih.gov/cdd

^5^ https://www.ncbi.nlm.nih.gov/blast

^6^ http://bioinformatics.psb.ugent.be/webtools/plantcare/html

^7^ https://phytozome.jgi.doe.gov/pz/portal

^8^ https://www.genoscope.cns.fr/brassicanapus/

^9^ http://www.genoscope.cns.fr/brassicanapus

^10^ http://sourceforge.net/projects/mev-tm4/files/mev-tm4/MeV%204.8.1/

^11^ http://selecton.tau.ac.il/

^12^ https://zhanglab.ccmb.med.umich.edu/I-TASSER/

^13^ https://brassica.biodb.org/

^14^ https://phytozome.jgi.doe.gov/pz/portal

^15^ http://www.genoscope.cns.fr/brassicanapus

^16^ https://www.ncbi.nlm.nih.gov/cdd

## Abbreviations

SHI/STY: SHORT INTERNODES/STYLISH
LRP1: LATERAL ROOT PRIMORDIUM1
SRS: SHI RELATED SEQUENCE3
GA: Gibberellin
NJ: Neighbor joining
LPAT4: Lysophosphatidic acid acyltransferase
MBOATs: Membrane-bound O-acyltransferases
PPR: Pentatricopeptide repeat
UBQ3: Ubiquitins
LDL: Low-density lipoprotein
CnB: Calcineurin B subunit-related
ABA: Abscisic acid
IAA: 3-Indoleacetic acid
6-BA: 6-Benzylaminopurine
