# Supplementary material for: Molecular evolutionary analysis of the SHI/STY gene family in land plants: A focus on the Brassica species
Source: Front Plant Sci. 2022 Aug 4;13:958964. doi: 10.3389/fpls.2022.958964 (PMC9386158; doi:10.3389/fpls.2022.958964)
Supplement: Supplementary file 1 [file Table_1.DOCX]

**Table S1** The primers of ten *BnSHI/STYs* for Quantitative Real Time PCR Analysis

| **Gene name** | **Forward Primer** | **Reverse Primer** |
| --- | --- | --- |
| BnaC09g44400D | ACATTAACTTCCTCGGAGACAG | GTCTGATGAGCACTCTTGAAGT |
| BnaA09g34300D | TCAACTTCCCTGCTAGAATCTC | GTAATTATAAAGCGCGTGGAGG |
| BnaC04g26320D | CAAGCGACTGTAACCATACATG | GAAAGACATGGAAAGGCTTTGT |
| BnaC01g31760D | GTTACGTGTTCAAAGGCATTCT | GATTTAGTCCTCAGCGGTGATA |
| BnaA07g12710D | TCACTGGTACACACTTCTTCTC | CTCCGTTCTACAAACCCTAGTT |
| BnaA01g19680D | ATGTTCCAGCATCAACAACATC | GCCGGAGATATCGAATTGACTA |
| BnaA03g59180D | AACAACAACAACGGATACCAAG | GGACCTTGATGATGTGGCTATA |
| BnaA09g43210D | ATAATGGGGAGAAGGTGTGAAG | TGAACTCACCTTTACACATCGA |
