# Supplementary material for: Molecular evolutionary analysis of the SHI/STY gene family in land plants: A focus on the Brassica species
Source: Front Plant Sci. 2022 Aug 4;13:958964. doi: 10.3389/fpls.2022.958964 (PMC9386158; doi:10.3389/fpls.2022.958964)
Supplement: Supplementary file 2 [file Table_2.DOCX]

| **Table S2 Subcellular location and physical and chemical properties of SHI family in land plants.** | | | | | | |
| --- | --- | --- | --- | --- | --- | --- |
| **Gene ID** | **Genomic Location** | **Protein Length** | **Molecular weight** | **pI** | **GRAVY** | **Localization** |
| AT1G19790.1 | Chr1:6838120-6840293 | 345 | 37928.37 | 6.19 | -0.809 | Nuclear (3.621) |
| AT1G75520.1 | Chr1:28351562-28353495 | 346 | 38553.96 | 6.67 | -0.968 | Nuclear (3.904) |
| AT2G18120.1 | Chr2:7876615-7877681 | 222 | 24879.36 | 8.08 | -0.981 | Nuclear (3.675) |
| AT2G21400.1 | Chr2:9158390-9159701 | 174 | 19813.72 | 9.39 | -0.56 | Nuclear (1.921); Extracellular (2.343) |
| AT3G51060.1 | Chr3:18964344-18966405 | 370 | 38352.13 | 7.13 | -0.441 | Nuclear (3.351) |
| AT3G54430.1 | Chr3:20147101-20148127 | 183 | 20326.32 | 9.77 | -0.538 | Nuclear (2.564) |
| AT4G36260.1 | Chr4:17155556-17157245 | 322 | 34144.16 | 6.48 | -0.776 | Nuclear (4.349) |
| AT5G12330.1 | Chr5:3987375-3989406 | 320 | 33617.81 | 8.77 | -0.621 | Nuclear (2.958) |
| AT5G33210.1 | Chr5:12465057-12465713 | 173 | 19542.68 | 9.42 | -0.426 | Nuclear (2.753) |
| AT5G66350.1 | Chr5:26504467-26506569 | 330 | 35289.75 | 8.52 | -0.732 | Nuclear (3.904) |
| BnaA01g01350D | chrC07:21603710-21605341 | 313 | 33741.48 | 5.82 | -0.897 | Nuclear (4.284) |
| BnaA01g19680D | chrC07:22808152-22809699 | 235 | 25203.93 | 9.13 | -0.715 | Nuclear (4.449) |
| BnaA02g17280D | chrA02:10389281-10390641 | 342 | 38108.74 | 6.8 | -0.859 | Nuclear (4.22) |
| BnaA03g59180D | chrA07:16733414-16734764 | 315 | 34159.28 | 7.66 | -0.856 | Nuclear (4.25) |
| BnaA04g04120D | chrA09:23636882-23637619 | 201 | 22303.12 | 9.41 | -0.728 | Nuclear (3.849) |
| BnaA06g13970D | chrA06:7408944-7410219 | 346 | 37911.25 | 6.19 | -0.794 | Nuclear (3.789) |
| BnaA07g01860D | chrC09:45250806-45252450 | 219 | 24394.89 | 7.02 | -0.896 | Nuclear (3.39) |
| BnaA07g12710D | chrC06:3329330-3330945 | 335 | 35368.78 | 8.24 | -0.633 | Nuclear (4.18) |
| BnaA07g21650D | chrA01:11065464-11066171 | 331 | 36698.38 | 7.67 | -0.759 | Nuclear (3.831) |
| BnaA09g31700D | chrC04:27700142-27701200 | 245 | 26245.1 | 9.26 | -0.669 | Nuclear (4.178) |
| BnaA09g34300D | chrA01:720686-722006 | 311 | 34360.92 | 9.49 | -0.443 | Nuclear (2.878) |
| BnaA09g43210D | chrA10:14277820-14279260 | 180 | 20709.69 | 9.69 | -0.703 | Nuclear (3.587) |
| BnaA10g20370D | chrC02:21599447-21600814 | 307 | 32740.9 | 8.6 | -0.7 | Nuclear (3.063) |
| BnaC01g02360D | chrA07:11402203-11403782 | 252 | 28088.73 | 6.04 | -0.884 | Nuclear (4.468) |
| BnaC01g31760D | chrC08:33420292-33421443 | 212 | 24399 | 5.56 | -0.346 | Cytoplasmic(1.425);Chloroplast(1.088) |
| BnaC02g11580D | chrC08:27029253-27030901 | 219 | 24366.88 | 7.57 | -0.909 | Nuclear (3.493) |
| BnaC02g24360D | chrC07:4955888-4956867 | 343 | 38294.91 | 6.65 | -0.877 | Nuclear (4.092) |
| BnaC04g26320D | chrA09:25167663-25169116 | 218 | 24686.05 | 9.69 | -0.75 | Nuclear (3.739) |
| BnaC06g22330D | chrA07:1553611-1554592 | 335 | 37219.97 | 7.67 | -0.795 | Nuclear (4.062) |
| BnaC06g43960D | chrC08:3408963-3410201 | 345 | 38310.84 | 6.2 | -0.871 | Nuclear (3.87) |
| BnaC07g03690D | chrC06:24385239-24386614 | 215 | 23906.64 | 8.3 | -0.74 | Nuclear (2.033); Extracellular (1.707) |
| BnaC07g05440D | chrA09:29990289-29991927 | 297 | 33120.13 | 6.37 | -0.658 | Nuclear (3.479) |
| BnaC07g15630D | chrA03:2229242-2230705 | 335 | 36713.3 | 6.72 | -0.773 | Nuclear (4.204) |
| BnaC07g16800D | chrC02:6883504-6884485 | 338 | 35665.24 | 8.45 | -0.619 | Nuclear (4.019) |
| BnaC08g25250D | chrA04:3030235-3031741 | 314 | 34792.54 | 9.22 | -0.396 | Extracellular (2.199) |
| BnaC08g35730D | chrC01:1234399-1235824 | 130 | 15283.6 | 10.06 | -0.93 | Nuclear (3.305) |
| BnaC08g48470D | chrC07:8725293-8727060 | 150 | 15066.46 | 4.56 | -0.047 | Nuclear (1.149); Extracellular (1.7) |
| BnaC09g44400D | chrC01:30654902-30658530 | 303 | 32217.39 | 8.76 | -0.665 | Nuclear (2.912) |
| Bol027153 | C07:730918-732081 | 374 | 42097.45 | 9.39 | -0.028 | PlasmaMembrane（4.490） |
| Bol027721 | C06:2197965-2199364 | 345 | 38264.75 | 6.2 | -0.88 | Nuclear（4.289） |
| Bol029004 | C01:1360266-1361692 | 307 | 33252.21 | 5.84 | -0.786 | Nuclear（3.833） |
| Bol041486 | C07:10924388-10925622 | 215 | 23906.64 | 8.3 | -0.74 | Nuclear（2.033） |
| Bol043504 | C09:35959931-35961525 | 406 | 42602.65 | 7.64 | -0.522 | Nuclear（3.224） |
| Brara.A00212.1.p | A01:1138580-1140497 | 313 | 33723.45 | 5.82 | -0.889 | Nuclear（4.294） |
| Brara.A02107.1.p | A01:12826349-12828847 | 357 | 37130.74 | 8.09 | -0.539 | Nuclear（3.709） |
| Brara.B00452.1.p | A02:2087987-2091585 | 310 | 32713.99 | 8.75 | -0.594 | Nuclear（2.799） |
| Brara.B02233.1.p | A02:14316117-14317993 | 342 | 38108.74 | 6.8 | -0.859 | Nuclear（4.221） |
| Brara.D00469.1.p | A04:3493803-3494943 | 189 | 21316.13 | 9.67 | -0.842 | Nuclear（2.758） |
| Brara.D01262.1.p | A04:11477374-11478577 | 190 | 21294.25 | 9.2 | -0.587 | Nuclear（3.686） |
| Brara.F01406.1.p | A06:7986199-7988021 | 346 | 37911.25 | 6.19 | -0.794 | Nuclear（3.790） |
| Brara.G00211.1.p | A07:1935819-1936800 | 219 | 24355.85 | 6.69 | -0.886 | Nuclear（3.191） |
| Brara.G01270.1.p | A07:13379487-13381535 | 335 | 35323.79 | 8.24 | -0.608 | Nuclear（4.131） |
| Brara.G02219.1.p | A07:19333727-19335557 | 331 | 36629.27 | 7.22 | -0.784 | Nuclear（3.775） |
| Brara.G03355.1.p | A07:25498918-25500882 | 345 | 38329.85 | 6.3 | -0.891 | Nuclear（3.866） |
| Brara.I03444.1.p | A09:32608503-32610780 | 367 | 37951.7 | 7.64 | -0.469 | Nuclear（3.054） |
| Brara.I03709.1.p | A09:34355881-34356990 | 191 | 21239.95 | 9.76 | -0.749 | Nuclear（3.209） |
| Brara.I04644.1.p | A09:39921216-39922854 | 130 | 14652.63 | 8.34 | -0.556 | Nuclear（3.072） |
| Brara.J02160.1.p | A10:16472614-16474886 | 401 | 42188.43 | 8.53 | -0.553 | Nuclear (3.686) |
| Brara.J02382.1.p | A10:17487413-17488714 | 232 | 26718.66 | 10.12 | -0.553 | Nuclear（2,028） |
| Brara.K01320.1.p | Scaffold58736:461150-462493 | 314 | 34045.18 | 7.66 | -0.848 | Nuclear（4.269） |
| Manes.01G179500.1.p | Chr01:35879593..35880785 | 359 | 37221.79 | 8.71 | -0.562 | Nuclear（3.291） |
| Manes.02G140000.1.p | Chr02:10784095..10785273 | 351 | 36498.94 | 7.23 | -0.527 | Nuclear（2.661） |
| Manes.04G159200.1.p | Chr04:35340555..35342899 | 268 | 29071.91 | 7.56 | -0.697 | Nuclear（3.773） |
| Manes.02G100100.1.p | Chr02:7796596..7802439 | 332 | 35513.27 | 6.44 | -0.548 | Nuclear（4.279） |
| Manes.05G153200.2.p | Chr05:26445827..26449889 | 306 | 33855.3 | 6.45 | -0.823 | Nuclear（3.757） |
| Manes.01G141500.1.p | Chr01:33287673..33292046 | 340 | 36337.13 | 7.53 | -0.596 | Nuclear（4.365） |
| Manes.04G085450.2.p | Chr04:28705198..28706865 | 209 | 22841.73 | 9.4 | -0.467 | Nuclear（2.667） |
| Manes.11G087500.1.p | Chr11:13950061..13951673 | 213 | 23048.99 | 8.98 | -0.35 | Nuclear（4.430） |
| Manes.18G018200.2.p | Chr18:1853520..1856773 | 312 | 34405.92 | 8.35 | -0.789 | Nuclear（3.469） |
| Manes.15G171600.1.p | Chr15:15394613..15402883 | 376 | 41495.31 | 7.57 | -0.718 | Nuclear（4.432） |
| R.communis000246 | 27742:9164..11472 forward | 344 | 35836.49 | 8.31 | -0.462 | Nuclear（2.237） |
| R.communis007187 | 30131:2101152..2103778 reverse | 280 | 30282.47 | 8.83 | -0.698 | Nuclear（4.227） |
| R.communis000131 | 29279:8685..10323 forward | 350 | 37860.54 | 6.65 | -0.77 | Nuclear（3.546） |
| R.communis014124 | 30170:2979704..2981115 reverse | 331 | 34744.24 | 8.34 | -0.853 | Nuclear（3.304） |
| R.communis007102 | 30131:1537108..1537981 reverse | 199 | 22113.88 | 8.64 | -0.588 | Nuclear（4.185） |
| R.communis000787 | 29628:227164..231717 forward | 366 | 41300.54 | 8.49 | -0.854 | Nuclear（4.084） |
| Potri.009G070800.3.p | Chr09:6973258..6976928 | 354 | 36705.38 | 8.73 | -0.458 | Nuclear（2.434） |
| Potri.001G276200.3.p | Chr01:28981194..28984054 | 358 | 37367.04 | 8.37 | -0.488 | Nuclear（2.173） |
| Potri.001G027700.1.p | Chr01:2099847..2103404 | 286 | 30976.42 | 9.06 | -0.659 | Nuclear（3.405） |
| Potri.003G196100.1.p | Chr01:28981194..28984054 | 286 | 31066.45 | 8.54 | -0.63 | Nuclear（4.629） |
| Potri.005G234200.1.p | Chr05:23265164..23267585 | 315 | 34218.58 | 8.35 | -0.757 | Nuclear（3.721） |
| Potri.002G028500.3.p | Chr02:1915522..1919306 | 294 | 32172.45 | 8.88 | -0.808 | Nuclear（3.866） |
| Potri.007G017500.1.p | Chr07:1337745..1340420 | 329 | 35644.55 | 6.65 | -0.613 | Nuclear（3.987） |
| Potri.005G118200.1.p | Chr05:8673014..8675431 | 344 | 37141.04 | 7.15 | -0.614 | Nuclear（4.126） |
| Potri.009G121600.3.p | Chr09:10181897..10184054 | 205 | 22843.91 | 9.13 | -0.574 | Nuclear（2.426） |
| Potri.004G160600.4.p | Chr04:18043562..18048493 | 198 | 21957.77 | 8.86 | -0.599 | Nuclear（3.405） |
| Potri.003G085901.1.p | Chr03:11247734..11253713 | 373 | 41888 | 8.77 | -0.803 | Nuclear（3.925） |
| Potri.001G148500.1.p | Chr01:12295731..12301520 | 373 | 41718.7 | 8.52 | -0.797 | Nuclear（3.934） |
| Medtr5g089750.1 | chr5:39017916..39020566 | 467 | 49225.01 | 8.34 | -0.595 | Nuclear（3.772） |
| Medtr3g014660.1 | chr3:4198129..4203654 | 329 | 35636.5 | 7.2 | -0.667 | Nuclear（3.347） |
| Medtr8g039110.1 | chr8:14512196..14513962 | 327 | 35781.87 | 7.92 | -0.66 | Nuclear（4.189） |
| Medtr8g076620.1 | chr8:32482155..32484876 | 331 | 36121.52 | 6.82 | -0.74 | Nuclear（3.819） |
| Medtr5g021130.1 | chr5:8100970..8103215 | 323 | 35563.04 | 5.91 | -0.692 | Nuclear（3.873） |
| Medtr3g112510.1 | chr3:52766534..52767953 | 294 | 32711.66 | 8.62 | -0.651 | Nuclear（4.048） |
| Medtr4g071110.1 | chr4:26794244..26795870 | 264 | 30126.09 | 8.75 | -0.533 | Nuclear（3.800） |
| Medtr0363s0040.1 | scaffold0363:16318..17438 | 245 | 27429.76 | 9.23 | -0.778 | Nuclear（3.802） |
| Medtr1g023230.1 | chr1:7417435..7419015 | 252 | 28225.74 | 8.56 | -1.114 | Nuclear（3.719） |
| Medtr0105s0070.1 | scaffold0105:22115..24983 | 86 | 9345.87 | 8.27 | 0.085 | Extracellular（4.068） |
| Medtr4g099070.1 | chr4:41015146..41023331 | 359 | 39685.22 | 5.9 | -0.805 | Nuclear（4.123） |
| GlymaLee.14G031700.1.p | Gm14:2633143..2636217 | 349 | 36900.78 | 8.55 | -0.571 | Nuclear（3.607） |
| GlymaLee.02G239200.1.p | Gm02:50128537..50131373 | 353 | 37225.95 | 8.55 | -0.651 | Nuclear（3.632） |
| GlymaLee.20G028400.1.p | Gm20:5679113..5683577 | 340 | 36080.48 | 7.26 | -0.663 | Nuclear (2.441);Extracellular (1.930) |
| GlymaLee.07G194800.1.p | Gm07:42675630..42679793 | 330 | 35295.62 | 6.54 | -0.681 | Nuclear（3.287） |
| GlymaLee.04G114100.1.p | Gm04:20161543..20167217 | 332 | 35377.43 | 8.48 | -0.486 | Nuclear（2.501） |
| GlymaLee.11G169700.1.p | Gm11:19279232..19284914 | 332 | 35702.82 | 8.78 | -0.513 | Nuclear（3.172） |
| GlymaLee.15G201600.1.p | Gm15:47682275..47687814 | 319 | 34731.27 | 7.27 | -0.73 | Nuclear（3.523） |
| GlymaLee.13G162400.1.p | Gm13:31270470..31275919 | 319 | 34173.86 | 8.23 | -0.581 | Nuclear（3.288） |
| GlymaLee.04G026600.1.p | Gm04:2264214..2266763 | 305 | 33185.68 | 6.39 | -0.589 | Nuclear（3.401） |
| GlymaLee.16G108100.1.p | Gm16:30490016..30493325 | 317 | 34670.81 | 6.62 | -0.825 | Nuclear（4.475） |
| GlymaLee.02G047600.1.p | Gm02:4774988..4778368 | 327 | 35389.6 | 6.96 | -0.766 | Nuclear（4.375） |
| GlymaLee.06G025300.1.p | Gm06:2205752..2208642 | 301 | 33032.53 | 7.62 | -0.672 | Nuclear（3.767） |
| GlymaLee.14G176700.1.p | Gm14:50435914..50438462 | 333 | 36073.57 | 6.82 | -0.696 | Nuclear（3.791） |
| GlymaLee.01G136300.1.p | Gm01:52612194..52619585 | 316 | 34300.87 | 6.06 | -0.642 | Nuclear（4.267） |
| GlymaLee.11G067300.1.p | Gm11:5502998..5505613 | 334 | 36133.91 | 6.22 | -0.641 | Nuclear（4.752） |
| GlymaLee.11G105900.1.p | Gm11:8781955..8783508 | 215 | 24179.33 | 9.15 | -0.695 | Nuclear（4.263） |
| GlymaLee.04G009100.1.p | Gm04:760178..761443 | 200 | 21976.44 | 8.87 | -0.663 | Nuclear（3.498） |
| GlymaLee.12G036500.1.p | Gm12:2877920..2879017 | 204 | 22692.53 | 8.62 | -0.66 | Nuclear（3.675） |
| GlymaLee.04G114100.4.p | Gm04:20161543..20167217 | 241 | 26170.23 | 8.58 | -0.672 | Extracellular（2.852） |
| GlymaLee.17G223100.1.p | Gm17:42320064..42322580 | 326 | 35594.13 | 6.82 | -0.738 | Nuclear（3.724） |
| Cucsa.087040.1 | scaffold00873:361047..362845 | 365 | 37662.42 | 7.15 | -0.417 | Nuclear (1.565); Extracellular (2.019) |
| Cucsa.138470.1 | scaffold01063:172185..174711 | 307 | 31146.62 | 8.9 | -0.338 | Nuclear（3.431） |
| Cucsa.307920.1 | scaffold02978:199558..200995 | 283 | 29868.14 | 8.89 | -0.551 | Nuclear（2.231） |
| Cucsa.043300.1 | scaffold00542:153220..156985 | 331 | 35897.29 | 7 | -0.769 | Nuclear（4.310） |
| Cucsa.201140.1 | scaffold01374:74326..76287 | 263 | 28663.74 | 6.13 | -0.746 | Nuclear（3.647） |
| Cucsa.159620.1 | scaffold01144:36973..38352 | 347 | 37386.3 | 6.45 | -0.607 | Nuclear（3,562） |
| Cucsa.342770.1 | scaffold03356:5049137..5050487 | 302 | 33808.18 | 7.2 | -0.855 | Nuclear（3,774） |
| Cucsa.345910.1 | scaffold03443:211327..212585 | 317 | 35198.91 | 7.1 | -0.737 | Nuclear（4.087） |
| Cucsa.321510.1 | scaffold03080:1301649..1308234 | 388 | 43201.45 | 8.93 | -0.865 | Nuclear（4.188） |
| Prupe.4G267200.1.p | Pp04:20095611..20098428 | 425 | 43709.44 | 8.1 | -0.467 | Nuclear（4.051） |
| Prupe.2G036500.1.p | Pp02:3943571..3946596 | 304 | 31982.79 | 8.56 | -0.496 | Extracellular（2.774） |
| Prupe.7G163600.1.p | Pp07:16853453..16859419 | 95 | 43309.9 | 6.45 | -0.654 | Nuclear（4.397） |
| Prupe.8G088300.1.p | Pp08:12137882..12139434 | 234 | 26063.32 | 8.99 | -0.632 | Nuclear（4.183） |
| Prupe.1G371500.1.p | Pp01:33860981..33863529 | 372 | 40034.22 | 5.8 | -0.934 | Nuclear（4.228） |
| Prupe.5G050200.1.p | Pp05:5322143..5329508 | 389 | 43122.46 | 8.02 | -0.747 | Nuclear（3.915） |
| MD03G1166900 | Chr03:22344770..22346671 | 414 | 42354.73 | 7.31 | -0.479 | Nuclear（4.122） |
| MD11G1186100 | Chr11:25245178..25247597 | 411 | 42575.05 | 7.73 | -0.477 | Nuclear（4.103） |
| MD02G1285800 | Chr02:34273862..34276419 | 347 | 37330.97 | 9.15 | -0.514 | Nuclear (2.237); Extracellular (2.060) |
| MD08G1021500 | Chr08:1551262..1553745 | 362 | 38491.12 | 6.38 | -0.714 | Nuclear（3.635） |
| MD15G1019200 | Chr15:1113802..1115731 | 360 | 38424 | 6.91 | -0.782 | Nuclear（3.540） |
| MD15G1250700 | Chr15:21081817..21084474 | 361 | 39675.73 | 7.06 | -0.758 | Nuclear（4.500） |
| MD05G1058700 | Chr05:10147513..10148466 | 232 | 25404.3 | 9.26 | -0.695 | Nuclear（4.224） |
| MD10G1066400 | Chr10:9092325..9093733 | 229 | 25523.44 | 9.36 | -0.725 | Nuclear（4.495） |
| MD02G1137000 | Chr02:11452119..11454065 | 372 | 40422.55 | 7.31 | -0.774 | Nuclear（4.369） |
| MD16G1267400 | Chr16:34263545..34268747 | 388 | 43036.44 | 9.16 | -0.81 | Nuclear（4.172） |
| MD06G1043400 | Chr06:5693744..5700360 | 387 | 43109.32 | 8.59 | -0.842 | Nuclear（4.015） |
| AL6G23130.t1 | scaffold_6:5003636..5006096 | 402 | 41663.63 | 8.6 | -0.515 | Nuclear（3.390） |
| AL5G31480.t1 | scaffold_5:15486707..15488911 | 369 | 38079.87 | 7.15 | -0.439 | Nuclear（3.576） |
| AL1G32500.t1 | scaffold_1:8391617..8393698 | 347 | 38029.5 | 6.04 | -0.751 | Nuclear（3.493） |
| AL2G35510.t1 | scaffold_2:16746229..16751400 | 344 | 38399.75 | 6.54 | -0.977 | Nuclear（3.608） |
| AL8G45180.t1 | scaffold_8:22783381..22786789 | 346 | 37511.3 | 6.41 | -0.74 | Nuclear（4.562） |
| AL7G14770.t1 | scaffold_7:1904136..190629 | 325 | 34515.46 | 6.24 | -0.812 | Nuclear（4.240） |
| AL3G51310.t1 | scaffold_3:22548520..22549821 | 206 | 22934.29 | 7.02 | -0.894 | Nuclear（2.856） |
| AL4G10590.t1 | scaffold_4:298441..299626 | 193 | 22033.94 | 9.35 | -0.797 | Nuclear（4.217） |
| AL5G35480.t1 | scaffold_5:17012658..17013693 | 183 | 20254.26 | 9.75 | -0.515 | Nuclear（2.902） |
| C.papaya169.52 | supercontig_169:568490..570382 | 329 | 35928.32 | 8.76 | -0.51 | Nuclear（3.003） |
| C.papaya140.14 | supercontig_140:310931..312224 | 302 | 33780.26 | 6.75 | -0.801 | Nuclear（3.358） |
| C.papaya6.196 | supercontig_6:1570516..1572024 | 321 | 34569.93 | 6.75 | -0.739 | Nuclear（3.991） |
| C.papaya150.46 | supercontig_150:442224..443656 | 247 | 27532.35 | 8.84 | -0.801 | Nuclear（3.968） |
| VIT_206s0009g03450.1 | chr6:16711149..16712324 | 361 | 37329.5 | 8.51 | -0.353 | Nuclear (2.406); Extracellular (1.872) |
| VIT_213s0067g02860.1 | chr13_random:1553224..1556674 | 318 | 33932.11 | 8.46 | -0.374 | Nuclear (1.901); Extracellular (1.656) |
| VIT_204s0023g02780.1 | chr4:19358407..19359850 | 344 | 36781.51 | 8.06 | -0.687 | Nuclear（3.465） |
| VIT_203s0038g00310.1 | chr3:288652..289505 | 198 | 21956.04 | 9.67 | -0.451 | Nuclear（4.470） |
| VIT_218s0001g13420.1 | chr18:11486983..11488357 | 320 | 35487.37 | 8.03 | -0.781 | Nuclear（3.799） |
| Sobic.003G429800.1.p | Chr03:73266878..73271822 | 360 | 36670.31 | 7.7 | -0.391 | Nuclear（3.609） |
| Sobic.009G122600.1.p | Chr09:47420997..47422772 | 305 | 31089.56 | 9.3 | -0.393 | Nuclear（3.411） |
| Sobic.002G274500.1.p | Chr02:65723462..65725735 | 327 | 33395.01 | 9.11 | -0.406 | Nuclear（3.248） |
| Sobic.007G179000.1.p | Chr07:61207201..61208802 | 312 | 32037.11 | 8.44 | -0.555 | Nuclear（3.975） |
| Sobic.010G261700.1.p | Chr10:59801732..59804530 | 345 | 34065.62 | 9.01 | -0.214 | Nuclear（3.103） |
| Sobic.002G275000.1.p | Chr02:65773219..65774647 | 240 | 24624.08 | 6.07 | -0.336 | Nuclear（3.993） |
| Zm00001d011843 | 8:162564728..162565898 | 389 | 39817.85 | 8.52 | -0.421 | Nuclear（3.308） |
| Zm00001d009971 | 8:93018883..93020443 | 298 | 30203.42 | 8.12 | -0.343 | Nuclear (2.271); Extracellular (1.594) |
| Zm00001d038081 | 6:147117118..147118645 | 285 | 29480.88 | 9.46 | -0.373 | Nuclear (2.261); Extracellular (1.570) |
| Zm00001d021285 | 7:147853085..147854863 | 321 | 32731.36 | 9.11 | -0.325 | Nuclear（3.049） |
| Zm00001d006209 | 2:201896426..201897959 | 324 | 33081.83 | 9.26 | -0.32 | Nuclear（3.221） |
| Zm00001d053208 | 4:220087907..220093065 | 296 | 30643.72 | 8.16 | -0.52 | Nuclear（3.606） |
| Zm00001d032040 | 1:210206508..210211370 | 302 | 31519.62 | 7.77 | -0.557 | Nuclear（3.542） |
| Zm00001d036426 | 6:87953854..87955348 | 331 | 33376.93 | 9.01 | -0.294 | Nuclear（3.214） |
| Zm00001d014762 | 5:61966309..61967872 | 339 | 33766.33 | 9.01 | -0.227 | Nuclear（3.001） |
| Zm00001d021290 | 7:148061213..148062014 | 221 | 23231.87 | 6.43 | -0.291 | Nuclear（2.803） |
| LOC_Os01g72490.1 | Chr1:42037166..42039622 | 340 | 34771.19 | 7.27 | -0.415 | Nuclear（3.780） |
| LOC_Os05g32070.1 | Chr5:18693816..18695153 | 330 | 33183.73 | 8.76 | -0.23 | Nuclear（3.022） |
| LOC_Os08g43410.1 | Chr8:27458306..27459934 | 302 | 30689.8 | 8.67 | -0.503 | Nuclear（3.497） |
| LOC_Os09g36160.1 | Chr9:20838366..20840292 | 315 | 32021.54 | 8,97 | -0.361 | Nuclear（3.642） |
| LOC_Os06g49830.1 | Chr6:30161498..30162653 | 359 | 34954.38 | 8,68 | -0.239 | Nuclear（3.329） |
| Bradi2g61120.1.p | Bd2:58058370..58064809 | 344 | 34820.11 | 8.14 | -0.424 | Nuclear（3.731） |
| Bradi2g26823.2.p | Bd2:25606595..25610317 | 228 | 23823.19 | 8.93 | -0.481 | Nuclear（2.592） |
| Bradi3g42197.1.p | Bd3:43673637..43675799 | 307 | 31496.41 | 8.66 | -0.624 | Nuclear（3.573） |
| Bradi4g36030.1.p | Bd4:41223067..41226813 | 343 | 34816.27 | 8.77 | -0.4 | Nuclear（4.152） |
| Bradi1g34990.1.p | Bd1:30633957..30636505 | 350 | 35013.3 | 8.17 | -0.364 | Nuclear（3.592） |
| S.moe59069 | scaffold_23:1898212..1899200 | 123 | 13259.06 | 9.53 | -0.567 | Nuclear（2.193） |
| S.moe409925 | scaffold_11:1840716..1843237 | 603 | 61827.21 | 8.34 | -0.485 | Nuclear（4.203） |
| S.moe59082 | scaffold_18:627271..628168 | 118 | 12859.65 | 8.86 | -0.343 | Nuclear（2.161） |
| S.moe59269 | scaffold_38:152892..153424 | 122 | 13484.09 | 9.35 | -0.684 | Nuclear（2.168） |
| Pp3c21_16440V3.1.p | Chr21:10399269..10402722 | 289 | 30504.15 | 8.89 | -0.582 | Nuclear（4.050） |
| Pp3c18_8920V3.1.p | Chr18:6302001..6306445 | 291 | 30867.65 | 8.89 | -0.559 | Nuclear（3.574） |
