# Supplementary material for: Molecular evolutionary analysis of the SHI/STY gene family in land plants: A focus on the Brassica species
Source: Front Plant Sci. 2022 Aug 4;13:958964. doi: 10.3389/fpls.2022.958964 (PMC9386158; doi:10.3389/fpls.2022.958964)
Supplement: Supplementary file 3 [file Table_3.DOCX]

**Table S3** Divergence time of Brassica gene pairs in *SHI/STY* gene family

| **Seq_1** | **Seq_2** | ***Ks*** | **Divergence time (million years)** |
| --- | --- | --- | --- |
| AT4G36260.1 | AT2G18120.1 | 1.264127 | 45.14738825 |
| AT1G19790.1 | AT1G75520.1 | 1.048785 | 37.45661529 |
| AT5G12330.1 | AT3G54430.1 | 3.166205 | 113.0787413 |
| BnaC02g24360D | BnaA02g17280D | 0.031278 | 1.11706433 |
| BnaA07g21650D | BnaC06g22330D | 0.027091 | 0.96753505 |
| BnaC07g15630D | BnaA06g13970D | 0.317103 | 11.32511797 |
| BnaA07g12710D | BnaC07g16800D | 0.067437 | 2.40845365 |
| BnaA01g19680D | BnaA09g31700D | 0.216222 | 7.72221789 |
| BnaA01g01350D | BnaC01g02360D | 0.062897 | 2.24632046 |
| BnaA07g01860D | BnaC02g11580D | 0.021303 | 0.76082883 |
| BnaC09g44400D | BnaA10g20370D | 0.098381 | 3.51359621 |
| BnaA09g34300D | BnaC08g25250D | 0.063783 | 2.27797983 |
| BnaC04g26320D | BnaA04g04120D | 0.214809 | 7.67175619 |
| BnaC07g05440D | BnaC01g31760D | 0.756488 | 27.01741571 |
| Brara.A02107.1.p | Brara.I03444.1.p | 0.254309 | 9.08245436 |
| Brara.K01320.1.p | Brara.A00212.1.p | 0.239698 | 8.56064802 |
| Brara.D01262.1.p | Brara.I04644.1.p | 0.376329 | 13.44033849 |
| Brara.I03709.1.p | Brara.D00469.1.p | 0.42729 | 15.26036481 |
| Brara.J02382.1.p | Brara.B02233.1.p | 1.516332 | 54.1547116 |
| Bol027721 | Bol039313 | 0.284932 | 10.17613149 |
