# Supplementary material for: Molecular evolutionary analysis of the SHI/STY gene family in land plants: A focus on the Brassica species
Source: Front Plant Sci. 2022 Aug 4;13:958964. doi: 10.3389/fpls.2022.958964 (PMC9386158; doi:10.3389/fpls.2022.958964)
Supplement: Supplementary file 4 [file Table_4.DOCX]

| **gene name** | **duplication mechanisms** |
| --- | --- |
| BnaA01g01350D | WGD or Segmental |
| BnaA01g19680D | WGD or Segmental |
| BnaA02g17280D | WGD or Segmental |
| BnaA03g59180D | Dispersed |
| BnaA04g04120D | WGD or Segmental |
| BnaA06g13970D | Dispersed |
| BnaA07g01860D | WGD or Segmental |
| BnaA07g12710D | WGD or Segmental |
| BnaA07g21650D | WGD or Segmental |
| BnaA09g31700D | WGD or Segmental |
| BnaA09g34300D | WGD or Segmental |
| BnaA09g43210D | WGD or Segmental |
| BnaA10g20370D | WGD or Segmental |
| BnaC01g02360D | WGD or Segmental |
| BnaC01g31760D | Dispersed |
| BnaC02g11580D | Dispersed |
| BnaC02g24360D | WGD or Segmental |
| BnaC04g26320D | WGD or Segmental |
| BnaC06g22330D | WGD or Segmental |
| BnaC06g43960D | Dispersed |
| BnaC07g03690D | WGD or Segmental |
| BnaC07g05440D | Dispersed |
| BnaC07g15630D | Dispersed |
| BnaC07g16800D | WGD or Segmental |
| BnaC08g25250D | WGD or Segmental |
| BnaC08g35730D | WGD or Segmental |
| BnaC08g48470D | Dispersed |
| BnaA01g38010D | WGD or Segmental |

**Table S4** The mechanisms of *BnSHI/STYs* duplication.
