# Supplementary figures and images for: Molecular evolutionary analysis of the SHI/STY gene family in land plants: A focus on the Brassica species
Source: Front Plant Sci. 2022 Aug 4;13:958964. doi: 10.3389/fpls.2022.958964 (PMC9386158; doi:10.3389/fpls.2022.958964)

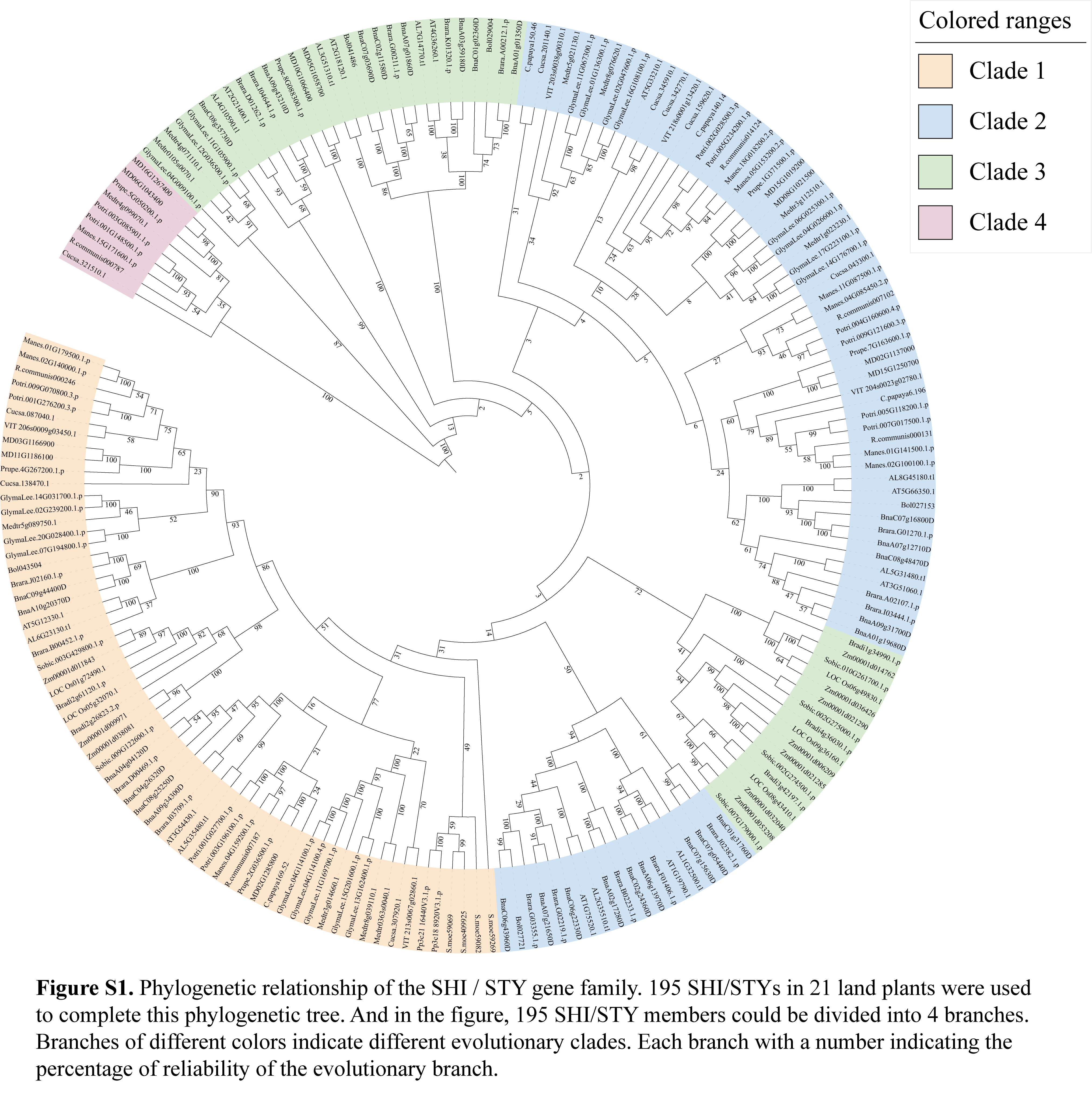

Supplement: Supplementary file 5 [file Image_1.TIF]

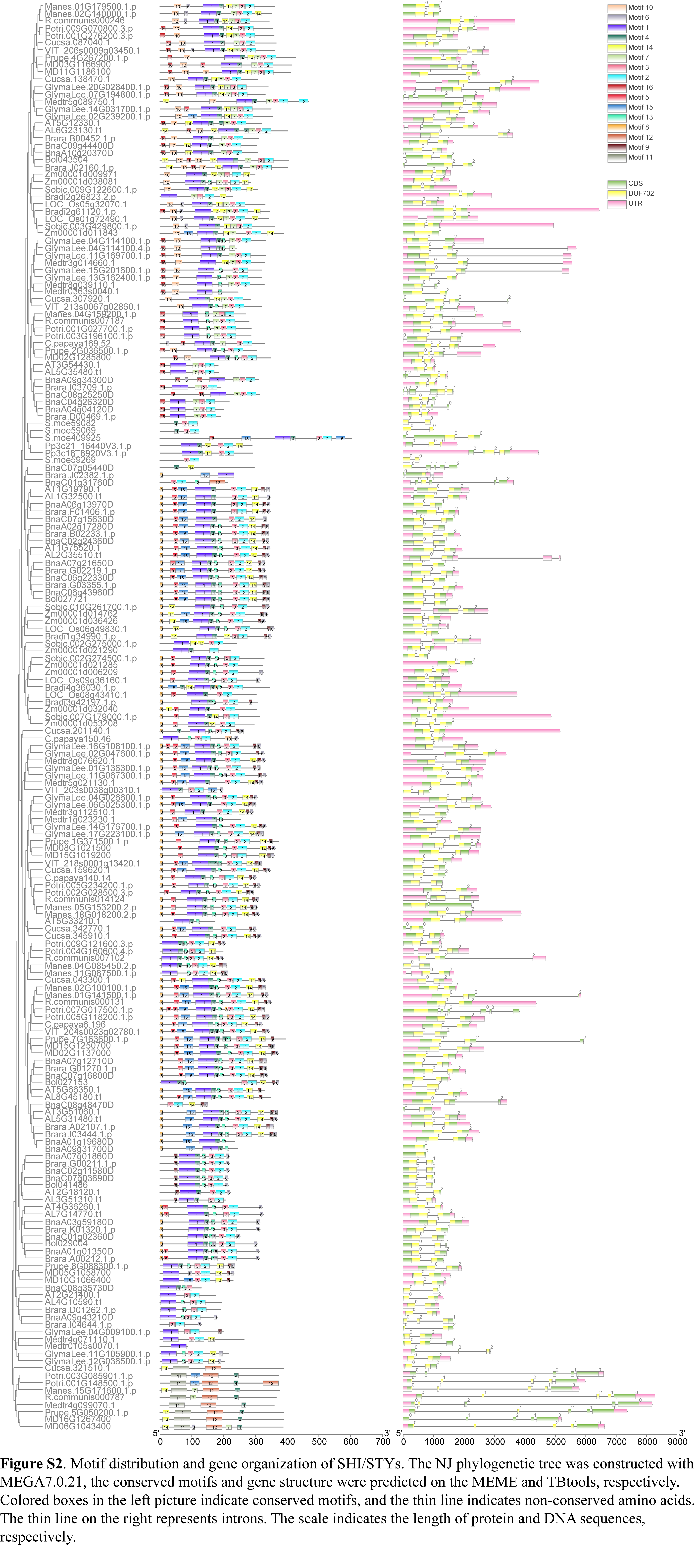

Supplement: Supplementary file 6 [file Image_2.TIF]
